# Efficacy and safety of *Ophiocordyceps sinensis* in the treatment of Hashimoto’s thyroiditis: a systematic review and meta-analysis

**DOI:** 10.3389/fphar.2023.1272124

**Published:** 2023-10-03

**Authors:** Maoying Wei, Wenxin Ma, Wenhua Zhang, Dan Yin, Yiting Tang, Weiyu Jia, Yijia Jiang, Churan Wang, Yanbing Gong

**Affiliations:** ^1^ Dongzhimen Hospital, Beijing University of Chinese Medicine, Beijing, China; ^2^ Centre for Evidence-Based Chinese Medicine, Beijing University of Chinese Medicine, Beijing, China

**Keywords:** Hashimoto’s thyroiditis, *Ophiocordyceps sinensis*, treatment, systematic review, meta-analysis

## Abstract

**Objective:** To evaluate the efficacy and safety of *Ophiocordyceps sinensis* (OS) preparations for the treatment of Hashimoto’s thyroiditis (HT).

**Methods:** We searched eight databases to collect randomized controlled trials (RCTs) of OS combined with a low-iodine diet or levothyroxine for HT. The search period was from inception to June 2023. Meta-analysis was performed using Revman 5.3 software after two evaluators independently screened the literature, extracted data, and evaluated the risk of bias of the included studies. The GRADE system was used to assess the certainty of evidence.

**Results:** A total of 14 RCTs involving 1,014 patients with HT were included. Meta-analysis showed that OS preparations combined with a low-iodine diet were more effective in reducing thyroid peroxidase antibody (TPOAb) [SMD = −3.81, 95% CI (−5.07, −2.54), *p* < 0.00001] and thyroglobulin antibody (TgAb) [SMD = −4.73, 95% CI (−6.86, −2.61), *p* < 0.00001] compared to a low-iodine diet. Compared with levothyroxine treatment alone, OS preparations combined with levothyroxine further reduced TPOAb [SMD = −2.04, 95% CI (−2.82, −1.26), *p* < 0.00001], TgAb [SMD = −2.01, 95% CI (−2.68, −1.33), *p* < 0.00001], tumor necrosis factor alpha (TNF-α) [SMD = −3.40, 95% CI (−5.66, −1.14), *p* = 0.003], interleukin-2 (IL-2) [SMD = −2.31, 95% CI (−3.98, −0.65), *p* = 0.006], and interleukin-6 (IL-6) [MD = −4.16, 95% CI (−6.17, −2.15), *p* < 0.0001], and elevated free thyroxine (FT4) [SMD = 1.34, 95% CI (0.59, 2.08), *p* = 0.0004], but no significant effect on free triiodothyronine (FT3) [SMD = 0.83, 95% CI (−0.12, 1.78), *p* = 0.09] and thyroid stimulating hormone (TSH) [SMD = −0.80, 95% CI (−1.71, 0.11), *p* = 0.08]. In terms of safety, three studies reported adverse reactions in 10 patients in each of the experimental and control groups.

**Conclusion:** OS preparations in combination with other treatments (low-iodine diet or levothyroxine) may decrease thyroid autoantibodies and inflammatory responses in patients with HT. In HT patients with hypothyroidism, the combination of the OS preparations with levothyroxine also improved FT4. However, the quality of the included studies was generally low. Moreover, the safety of OS preparations remains unclear. Therefore, more high-quality, multicenter, large-sample RCTs are needed in the future to validate the efficacy and safety of OS preparations.

**Systematic Review Registration:** https://www.crd.york.ac.uk/prospero, identifier CRD42023432663

## 1 Introduction

Hashimoto’s thyroiditis (HT), also known as chronic lymphocytic thyroiditis, is a common clinical autoimmune thyroid disease and the leading cause of hypothyroidism. In recent years, the prevalence of HT has increased every year as environmental factors have changed. The overall global prevalence of HT in adults is 7.5 (95% CI: 5.7%–9.6%). The risk for women is approximately four times higher than for men (Hu et al., 2022a). HT onset is insidious and progresses slowly. Its early clinical manifestations are often atypical until it progresses to permanent hypothyroidism. A growing number of studies have found that HT increases the risk of tumors (Hu et al., 2022b), polycystic ovary syndrome (Ho et al., 2020), new-onset systemic lupus erythematosus (Lin et al., 2023), and kidney transplant failure (Sigman et al., 2023). Chen et al. (2015) demonstrated a higher risk of coronary heart disease in HT patients compared with non-HT controls, with an adjusted hazard ratio of 1.44 (95% CI: 1.05–1.99).

The interaction of genetic susceptibility and environmental factors is a key factor in the breakdown of immune tolerance and the progression of HT. Currently, there is still a lack of specific treatment for HT in clinical practice. Selenium preparations, immunomodulatory therapies, and thyroid hormone replacement therapies for hypothyroid patients are the main interventions at this stage. However, most people with normal thyroid function and only elevated antibodies remain in the passive observation phase. *Ophiocordyceps sinensis* (OS, also called *Cordyceps sinensis*) is the dried complex of ascospores and larval carcasses of the fungus *C. sinensis* (BerK.) Sacc. parasitized on the larvae of the bat moth family. OS contains a variety of chemical components, including nucleosides, polysaccharides, proteins, amino acids, steroids, and polypeptides (Liu et al., 2015). Modern studies have shown that OS and its extracts have a wide range of pharmacological activities, such as immunomodulation (Li and Ren, 2017), antitumor (Li et al., 2020; Liu et al., 2022), antidepressant (Zhang et al., 2022), anti-hepatic fibrosis (Peng et al., 2016), nephroprotection (Liu et al., 2022; Zhang et al., 2023), cardioprotection (Wu et al., 2018), hypoglycemia, and hypolipidemia (Liu et al., 2019). Chinese patent medicines such as Bailing capsule, Jinshuibao capsule, Bailing tablet, etc. are OS preparations included in the Chinese Pharmacopoeia (2020 edition). Several clinical studies have demonstrated that these OS preparations reduce thyroid peroxidase antibody (TPOAb) and thyroglobulin antibody (TgAb) titers and improve autoimmune responses in HT patients (Liu et al., 2016; Li, 2019; Tan, 2021). However, most of these studies were small-sample, single-center clinical trials. This study systematically evaluates the efficacy and safety of OS preparations combined with a low-iodine diet or levothyroxine in the treatment of HT on the basis of existing literature, with a view to providing more reliable evidence for its clinical application.

## 2 Materials and methods

### 2.1 Protocol and registration

This meta-analysis was reported in strict accordance with the Preferred Reporting Items for Systematic Reviews and Meta-Analyses (PRISMA) guidelines (Page et al., 2021), as shown in Supplementary Table S1. The protocol of this study has been registered in the International Prospective Registry of Systematic Reviews (PROSPERO) (CRD42023432663). Ethical approval was not required to conduct this study, as all data were derived from published studies.

### 2.2 Inclusion criteria

#### 2.2.1 Type of studies

Randomized controlled trials (RCTs).

#### 2.2.2 Participants

Participants were diagnosed with Hashimoto’s thyroiditis, with or without hypothyroidism. There were no restrictions on age, gender, nationality, race, or duration of HT.

#### 2.2.3 Interventions

The experimental group was treated with OS preparations (e.g., Bailing capsule, Bailing tablet, Jinshuibao capsule, Jinshuibao tablet) combined with a low-iodine diet or levothyroxine (LT4). The control group was treated with a low-iodine diet or LT4. The low-iodine diet regimen and the type and dose of LT4 were the same in both groups. There are no restrictions on the dosage form, dose, or duration of OS preparations. The route of administration is oral.

#### 2.2.4 Outcomes

The primary outcomes included TPOAb, TgAb, free triiodothyronine (FT3), free thyroxine (FT4), and thyroid stimulating hormone (TSH). The secondary outcomes included tumor necrosis factor alpha (TNF-α), interleukin-2 (IL-2), and interleukin-6 (IL-6). Additional outcomes were adverse events.

### 2.3 Exclusion criteria

1) Full-text documents are not available. 2) Literature with missing data or clearly incorrect data. 3) There are no relevant outcome indicators in the literature. 4) Animal experiments, reviews, case reports, meta-analysis, etc. 5) RCTs published in languages other than Chinese or English. 6) Only the most recent and complete data were selected for duplicate published studies.

### 2.4 Search strategy

We searched PubMed, Cochrane Library, EMBASE, Web of Science, China National Knowledge Infrastructure (CNKI), Wanfang Data, VIP, and SinoMed databases to collect all RCTs of OS preparations for HT. The search period was from inception to 6 June 2023. The key search terms were “Hashimoto’s thyroiditis, or Hashimoto thyroiditis, or chronic lymphocytic thyroiditis, or autoimmune thyroiditis, or Hashimoto’s disease,” and “OS, or Bailing, or Jinshuibao.” According to the search characteristics of each database, multiple matching methods such as subject terms, free words, keywords, and abstracts are selected for systematic search to ensure the comprehensiveness of the search. The search details were provided in Supplementary Table S2.

### 2.5 Study selection and data extraction

Endnote software (version X9.1) was used for literature organization and classification. Primary screening was performed by abstract and title information. The full text was downloaded when the abstract and title did not provide sufficient inclusion and exclusion information. The literature was re-screened strictly according to the inclusion and exclusion criteria after reading through the entire text. We created Excel literature extraction tables. Extracted information included article author, publication date, sample size, patient baseline information, randomized sequence generation method, experimental and control group interventions (type, dose, administration, duration), outcomes, adverse events, etc. If outcomes were obtained at different doses of OS preparations, we only extracted the data for the highest dose group. Study selection and data extraction were done independently by two reviewers, and any disagreement was decided by a joint discussion involving a third reviewer.

### 2.6 Risk of bias assessment

The quality of the included studies was assessed using the Cochrane risk of bias tool (Higgins et al., 2011). Sources of bias included random sequence generation, allocation concealment, blinding of participants and personnel, blinding of outcome assessment, incomplete outcome data, selective reporting, and other bias. The final results were classified into three levels: “low risk”, “unclear risk”, and “high risk”. The risk of bias was assessed independently by two reviewers. Any disagreement was resolved by joint discussion with a third reviewer.

### 2.7 Statistical analysis

The data analysis was performed using Review Manager software (version 5.3, Copenhagen: The Nordic Cochrane Center, The Cochrane Collaboration, 2014). The outcomes included in this study were all continuous variables. In the case of different units or measurement methods for continuous variables, the standardized mean difference (SMD) was used instead of the mean difference (MD) as the combined effect indicator. Point estimates and 95% confidence intervals (CIs) were calculated for each effect size.

Heterogeneity among the outcomes of the included studies was analyzed using the Cochrane Q test, while the magnitude of heterogeneity was determined quantitatively in combination with I^2^ (Higgins and Thompson, 2002). When *p* ≥ 0.1 and I^2^ < 50%, it indicates that there is no statistically significant heterogeneity in each outcome, and meta-analysis was performed using a fixed-effects model. When *p* < 0.1 and I^2^>50% indicated statistical heterogeneity in each outcome, the source of heterogeneity was further analyzed. A meta-analysis was performed using a random-effects model after excluding the effect of significant clinical heterogeneity. Meta-analysis was performed at a test level of α = 0.05. Significant clinical heterogeneity was addressed using methods such as subgroup analysis, sensitivity analysis, or descriptive analysis only. This study was proposed to conduct a subgroup analysis for the type of OS preparations (Baling capsule or Jinshuibao capsule), duration of intervention, dose, etc. Sensitivity analysis was performed by the leave-one-out method. When the number of studies in the meta-analysis was 10 or more, the Egger’s test was performed using Stata software (version 16, The Stata Corporation, College Station, Texas, United States) to assess publication bias. If *p* < 0.05, it indicated that the study had publication bias.

### 2.8 Certainty assessment of evidence

The Grading of Recommendations, Assessment, Development, and Evaluations (GRADE) system was used to evaluate the quality of evidence for outcomes (Atkins et al., 2004). Levels of evidence were rated according to the risk of bias, inconsistency, indirectness, imprecision, and publication bias. The quality of the evidence was categorized into four levels: high, moderate, low, and very low.

## 3 Results

### 3.1 Study selection

**FIGURE 1:**
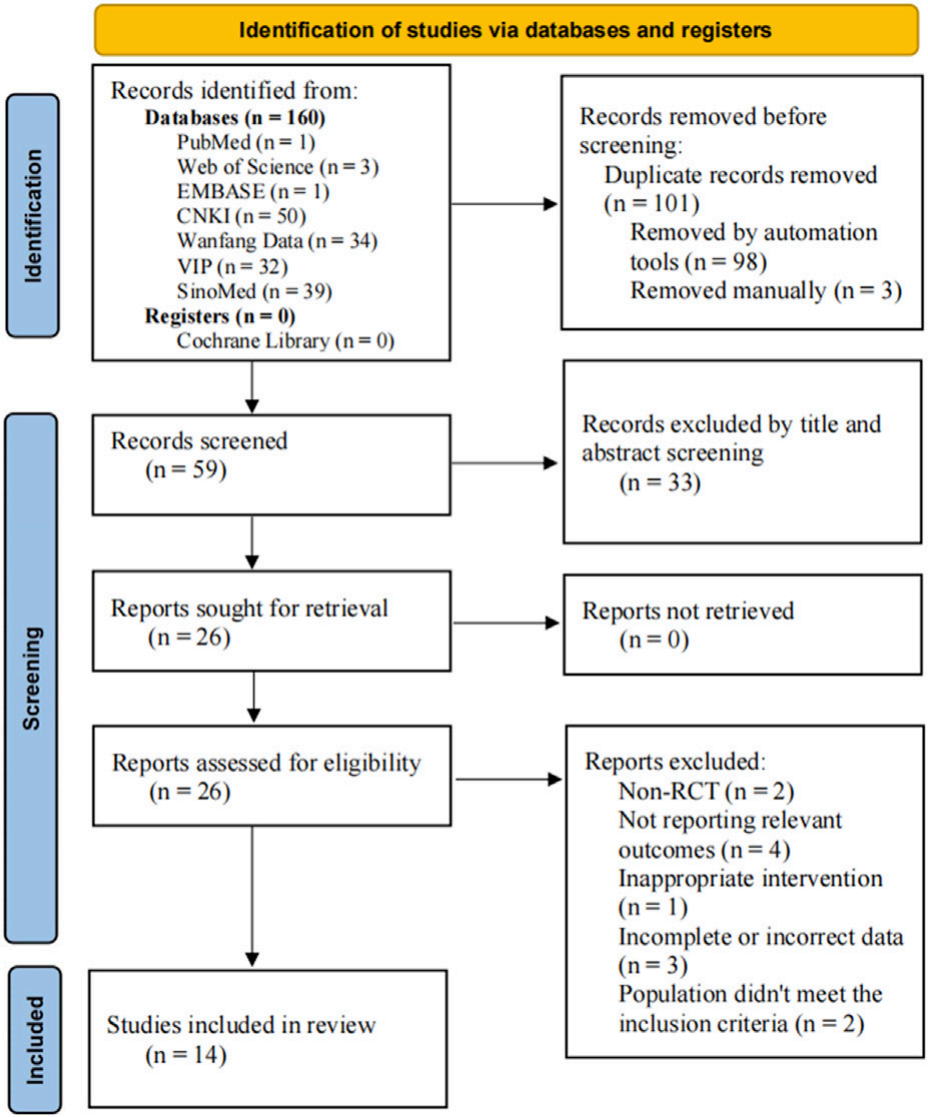
PRISMA flow diagram of record selection process.

A total of 160 articles were retrieved in this study, including 155 articles in Chinese and five articles in English. After the removal of 101 duplicates by EndNote X9.1 and manually, 59 articles were left for further examination. After screening the titles and abstracts, 33 articles were removed for various reasons: Meta-analysis (*n* = 2), animal experiments (*n* = 3), reviews (*n* = 2), improper intervention (*n* = 16), retrospective study (*n* = 2), not HT (*n* = 2), and non-RCT (*n* = 6). 26 RCTs were examined in full text, of which 14 met the inclusion criteria and 12 were excluded for the following reasons: non-RCT (*n* = 2), not reporting relevant outcomes (*n* = 4), inappropriate intervention (*n* = 1), incomplete or incorrect data (*n* = 3) and population did not meet the inclusion criteria (*n* = 2). Finally, 14 studies were included for systematic review and meta-analysis. The screening process is summarized in the flow diagram in Figure 1.

### 3.2 Study inclusion characteristics

A total of 1014 HT patients were included in the 14 RCTs, including 339 patients with normal thyroid function and 675 patients with hypothyroidism. These trials were conducted in mainland China from 2015 to 2021, of which 13 were published in Chinese (Zhang, 2015; Aisha, 2016; Li, 2017; Yang et al., 2018a; Yang et al., 2018b; Kang and Piao, 2019; Bai et al., 2020; Jin and Wang, 2020; Zhu et al., 2020; Tan, 2021; Wang et al., 2021; Xue et al., 2021; Zhan and Chen, 2021) and one in English (He et al., 2016). There were 523 and 491 patients in the experimental and control groups, respectively. The minimum sample size for inclusion in the trial was 47 cases, and the maximum sample size was 120 cases. There were two OS preparations used in the 14 RCTs: Bailing capsule and Jinshuibao capsule. Supplementary Table S3 presents details of these OS preparations. The duration of the intervention ranged from 8 weeks to 28 weeks. 5 RCTs compared OS preparations combined with a low-iodine diet to a low-iodine diet, of which 4 RCTs investigated Bailing capsule (Zhang, 2015; Li, 2017; Zhu et al., 2020; Xue et al., 2021) and one RCT investigated Jinshuibao capsule (Tan, 2021). 9 RCTs compared OS preparations combined with LT4 to LT4, of which 7 RCTs investigated Bailing capsule (Aisha, 2016; He et al., 2016; Yang et al., 2018a; Yang et al., 2018b; Jin and Wang, 2020; Wang et al., 2021; Zhan and Chen, 2021) and 2 RCTs investigated Jinshuibao capsule (Kang and Piao, 2019; Bai et al., 2020). The basic characteristics of the included studies are shown in Table 1.

**TABLE 1:** Basic characteristics of included studies.

Study ID	Sample size(E/C)	Age(year)		Male/Female		Intervention(s)	Comparators	Treatment duration(week)	With hypothyroidism	Outcomes
		E	C	E	C					
Zhang (2015)	28/28	32.0 ± 5.2		3/53		BLC 0.4g tid + LID	LID	24	not	①②③④⑤
Li et al. (2017)	48/48	48 ± 5		24/24	25/23	BLC 2.5g tid + LID	LID	12	not	①②③④⑤
Zhu et al. (2020)	30/30	35.07 ± 1.50	34.73 ± 1.45	0/30	0/30	BLC 1.5g tid + LID	LID	24	not	①②③④⑤
Tan (2021)	26/21	28.0 ± 5.7		6/41		JSBC 1.5g tid + LID	LID	28	not	①②③④⑤⑨
Xue et al. (2021)	40/40	41.47 ± 4.15	41.25 ± 4.13	20/20	18/22	BLC 1.0g tid + LID	LID	12	not	①③
Aisha (2016)	30/30	40.26 ± 8.17	40.71 ± 8.35	1/29	1/29	BLC 2.5g bid + LT4	LT4	24	yes	①②③④⑤
He et al. (2016)	39/17	41.40 ± 13.76	43.82 ± 12.48	11/28	7/10	BLC 2.0g tid + LT4	LT4	24	yes	①②③④⑤
Yang et al. (2018a)	35/30	NA		NA	NA	BLC 2.0g tid + LT4	LT4	24	yes	①②③④⑤⑨
Yang et al. (2018b)	40/40	35.5 ± 9.8	35.9 ± 11.2	8/32	7/33	BLC 3.0g tid + LT4	LT4	24	yes	①②③④⑤⑨
Kang and Piao (2019)	35/35	47.14 ± 8.83	48.32 ± 9.12	3/32	5/30	JSBC 0.99g tid + LT4	LT4	24	yes	①②③④⑤⑥⑦⑨
Bai et al. (2020)	40/40	43.79 ± 3.46	43.68 ± 4.82	22/18	24/16	JSBC 0.99g bid + LT4	LT4	8	yes	①②③④⑤⑥⑧⑨
Jin and Wang (2020)	60/60	43.28 ± 6.87		22/98		BLC 2.5g tid + LT4	LT4	12	yes	①②③④⑤
Wang et al. (2021)	32/32	38.69 ± 7.88	37.85 ± 7.33	9/23	10/22	BLC 2.5g tid + LT4	LT4	12	yes	③④⑤
Zhan and Chen (2021)	40/40	32.56 ± 5.78	32.98 ± 3.01	11/29	13/27	BLC 1.0g tid + LT4	LT4	8	yes	①②③④⑤⑥⑦⑧⑨

E, experimental group; C, control group; BLC, bailing capsule; JSBC, jinshuibao capsule; LID, low-iodine diet; LT4, levothyroxine; ①free triiodothyronine (FT3); ②free thyroxine (FT4); ③thyroid stimulating hormone (TSH); ④thyroid peroxidase antibody (TPOAb); ⑤thyroglobulin antibody (TgAb); ⑥tumor necrosis factor alpha (TNF-α); ⑦interleukin-2 (IL-2); ⑧interleukin-6 (IL-6); ⑨adverse events.

### 3.3 Quality assessment of included studies

**FIGURE 2:**
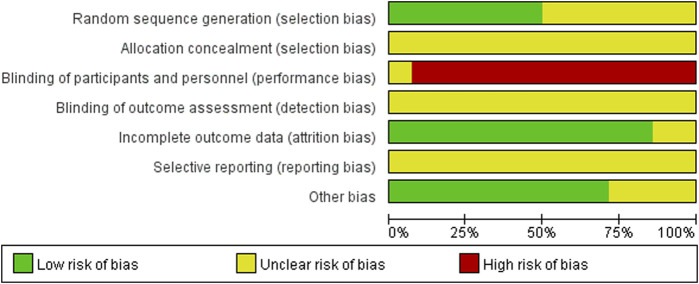
Risk of bias summary.

The 14 included studies referred to random allocation, with seven studies reporting specific grouping methods, including five studies using the random number table method (Kang and Piao, 2019; Bai et al., 2020; Zhu et al., 2020; Xue et al., 2021; Zhan and Chen, 2021), 1 study using the lottery method (Li, 2017), and 1 study using the random number residual method (Yang et al., 2018b). None of the studies reported allocation concealment methods. One study mentioned blinding but did not describe the details (He et al., 2016). None of the 14 studies mentioned the use of blinding for outcome assessment. Twelve studies had no missing data or a loss of follow-up rate of less than 10% (Zhang, 2015; Li, 2017; Yang et al., 2018a; Yang et al., 2018b; Kang and Piao, 2019; Bai et al., 2020; Jin and Wang, 2020; Zhu et al., 2020; Tan, 2021; Wang et al., 2021; Xue et al., 2021; Zhan and Chen, 2021). Two studies did not describe the number or reasons for the loss of follow-up (Aisha, 2016; He et al., 2016). All included studies showed an unclear bias risk in ‘selective reporting’ because their protocols were not registered in advance, so we could not judge whether all pre-determined outcomes were reported. The male-to-female sex ratio of the patients in four studies did not correspond to clinical reality (Li, 2017; Yang et al., 2018a; Bai et al., 2020; Xue et al., 2021). Therefore, other biases may exist. The risk of bias assessment of the included studies is shown in Figure 2.

### 3.4 Meta-analysis results

#### 3.4.1 Primary outcomes

(1) Thyroid autoantibodies

**FIGURE 3:**
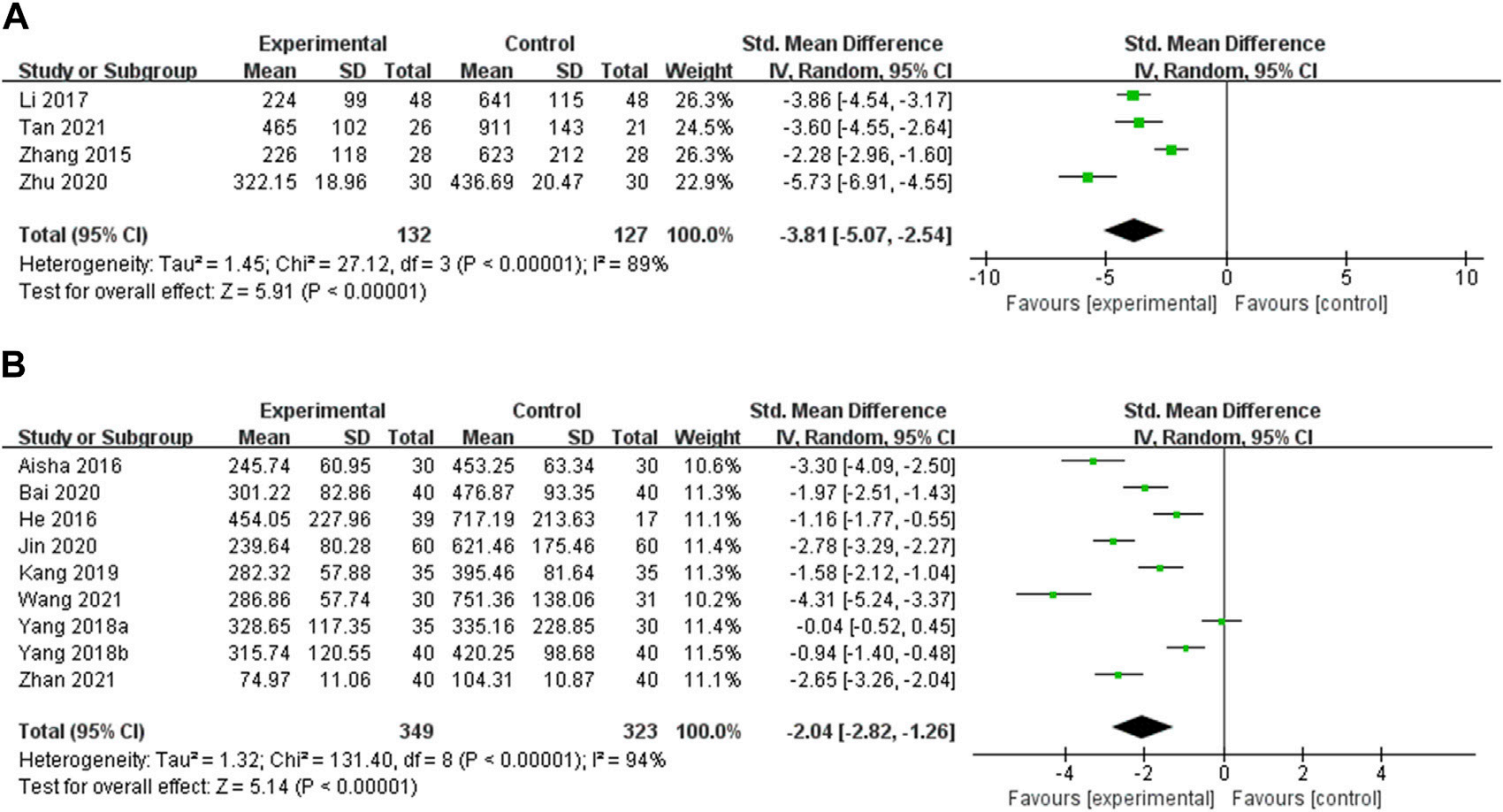
Meta-analysis results for the effect of OS preparations on TPOAb in HT patients. **(A)** OS combined with a low-iodine diet vs. a low-iodine diet; **(B)** OS combined with LT4 vs. LT4.

TPOAb TPOAb was reported in 13 studies. Four studies compared OS preparations combined with a low-iodine diet to a low-iodine diet (Zhang, 2015; Li, 2017; Zhu et al., 2020; Tan, 2021). Heterogeneity analysis showed statistical heterogeneity among the studies (*p* < 0.00001, I^2^ = 89%). Therefore, meta-analysis was performed using random effects models. The results showed that the experimental group was more effective in reducing TPOAb than the control group [SMD = −3.81, 95% CI (−5.07, −2.54), *p* < 0.00001] (Figure 3A). Subgroup analyses could not explain the heterogeneity (Supplementary Figure S1, Supplementary Table S4). Nine studies compared OS preparations combined with LT4 to LT4 (Aisha, 2016; He et al., 2016; Yang et al., 2018a; Yang et al., 2018b; Kang and Piao, 2019; Bai et al., 2020; Jin and Wang, 2020; Wang et al., 2021; Zhan and Chen, 2021). Random effects models were used to analyze pooled data [SMD = −2.04, 95% CI (−2.82, −1.26), *p* < 0.00001] due to significant heterogeneity (*p* < 0.00001, I^2^ = 94%). Meta-analysis suggested that OS preparations combined with LT4 were more beneficial than LT4 in reducing TPOAb (Figure 3B). Subgroup analyses showed significantly lower heterogeneity in the Jinshuibao capsule group. This suggests that the type of OS preparation may be the source of heterogeneity (Supplementary Figure S2, Supplementary Table S4).

**FIGURE 4:**
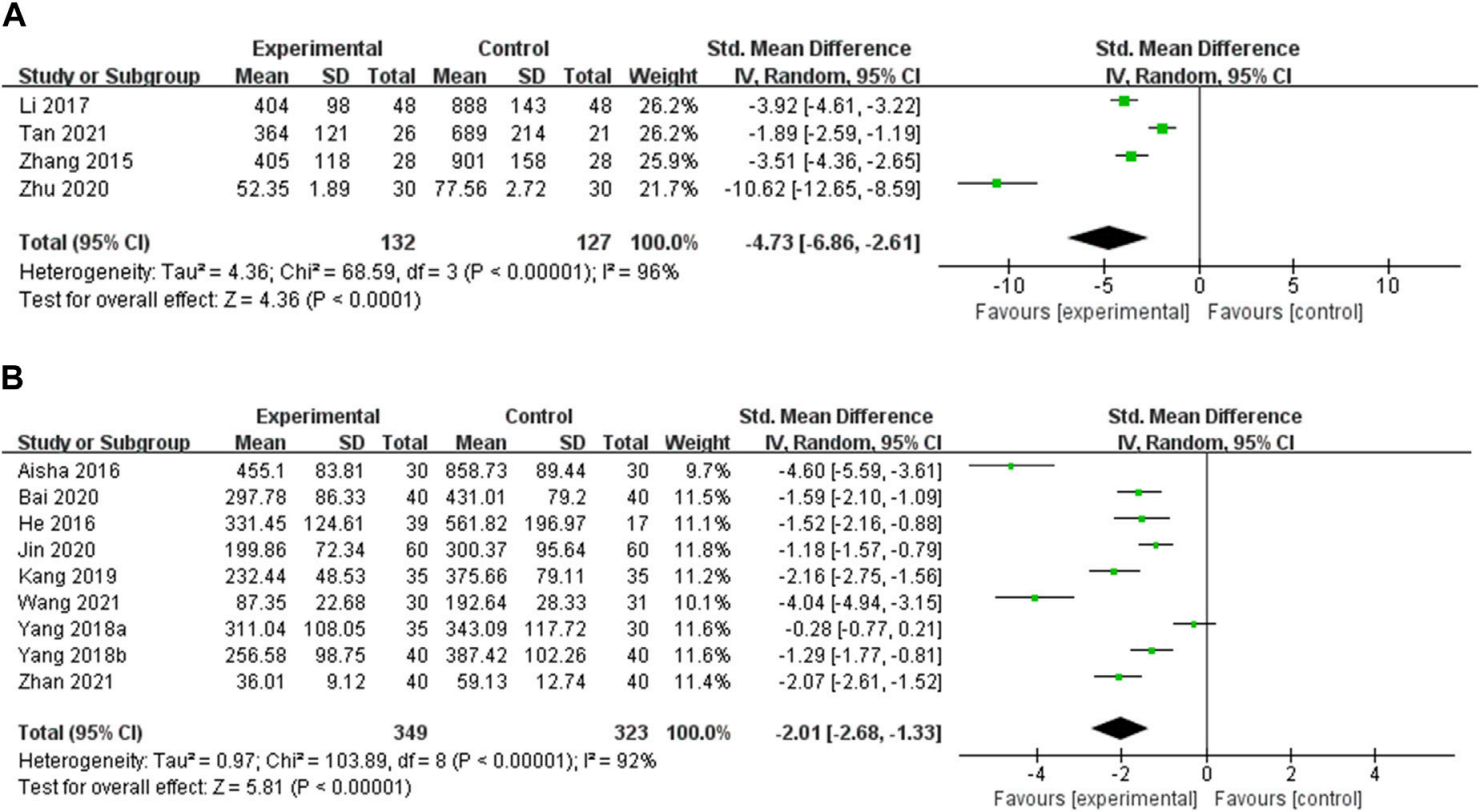
Meta-analysis results for the effect of OS preparations on TgAb in HT patients. **(A)** OS combined with a low-iodine diet vs. a low-iodine diet; **(B)** OS combined with LT4 vs. LT4.

TgAb TgAb was reported in 13 studies. Four studies compared OS preparations combined with a low-iodine diet to a low-iodine diet (Zhang, 2015; Li, 2017; Zhu et al., 2020; Tan, 2021). Heterogeneity analysis showed a high degree of heterogeneity among the studies (*p* < 0.00001, I^2^ = 96%), so meta-analysis was performed using random effects models. The results showed that the experimental group was more effective in reducing TgAb than the control group [SMD = −4.73, 95% CI (−6.86, −2.61), *p* < 0.00001] (Figure 4A). Heterogeneity persisted despite subgroup analyses by duration of intervention, type of OS preparation, and dose (Supplementary Figure S3, Supplementary Table S4). Nine studies compared OS preparations combined with LT4 to LT4 (Aisha, 2016; He et al., 2016; Yang et al., 2018a; Yang et al., 2018b; Kang and Piao, 2019; Bai et al., 2020; Jin and Wang, 2020; Wang et al., 2021; Zhan and Chen, 2021). Random effects models were used for meta-analysis due to the high heterogeneity among studies (*p* < 0.00001, I^2^ = 92%). The results showed that the experimental group was more effective in reducing TgAb than the control group [SMD = −2.01, 95% CI (−2.68, −1.33), *p* < 0.00001] (Figure 4B). Heterogeneity remained after doing a subgroup analysis of intervention duration. Heterogeneity was substantially reduced in the Jinshuibao capsule group and the group with a dose of no more than 3 g/d. These suggest that the type and dose of OS preparation may be the source of heterogeneity (Supplementary Figure S4, Supplementary Table S4).

(2) Thyroid function

FT3 was reported in 13 studies (Zhang, 2015; Aisha, 2016; He et al., 2016; Li, 2017; Yang et al., 2018a; Yang et al., 2018b; Kang and Piao, 2019; Bai et al., 2020; Jin and Wang, 2020; Zhu et al., 2020; Tan, 2021; Xue et al., 2021; Zhan and Chen, 2021). FT4 was reported in 12 studies (Zhang, 2015; Aisha, 2016; He et al., 2016; Li, 2017; Yang et al., 2018a; Yang et al., 2018b; Kang and Piao, 2019; Bai et al., 2020; Jin and Wang, 2020; Zhu et al., 2020; Tan, 2021; Zhan and Chen, 2021). TSH was reported in 14 studies (Zhang, 2015; Aisha, 2016; He et al., 2016; Li, 2017; Yang et al., 2018a; Yang et al., 2018b; Kang and Piao, 2019; Bai et al., 2020; Jin and Wang, 2020; Zhu et al., 2020; Tan, 2021; Wang et al., 2021; Xue et al., 2021; Zhan and Chen, 2021). Subjects in these studies included both normal thyroid function and hypothyroid HT patients. FT3, FT4, and TSH were always within the normal range during the trial in HT patients with normal thyroid function. Therefore, their slight fluctuations have no actual clinical significance. In this study, we only analyzed the effect of adjuvant therapy with OS preparations on thyroid function in patients with HT combined with hypothyroidism.

**FIGURE 5:**
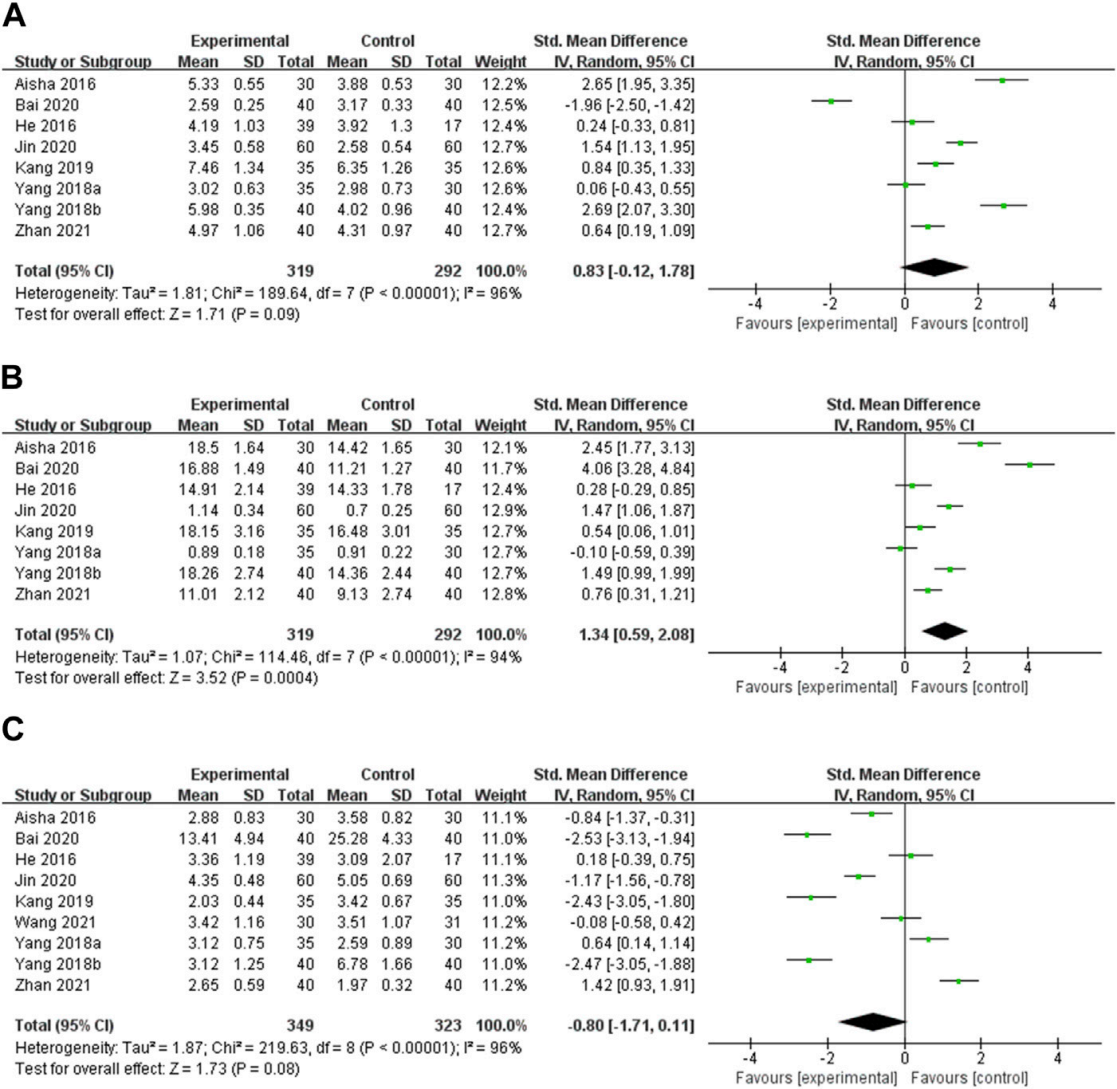
Meta-analysis results for the effect of OS preparations on thyroid function in HT patients with hypothyroidism. **(A)** FT3; **(B)** FT4; **(C)** TSH.

**FT3** Eight studies reported the effect of OS preparations on FT3 in HT patients with hypothyroidism (Aisha, 2016; He et al., 2016; Yang et al., 2018a; Yang et al., 2018b; Kang and Piao, 2019; Bai et al., 2020; Jin and Wang, 2020; Zhan and Chen, 2021). Heterogeneity analysis showed significant heterogeneity among the studies (*p* < 0.00001, I^2^ = 96%). Hence, meta-analysis was performed using random effects models. The results showed that there was no statistically significant difference in FT3 between the experimental and control groups [SMD = 0.83, 95% CI (−0.12, 1.78), *p* = 0.09] (Figure 5A). Heterogeneity persisted after doing subgroup analyses of intervention duration, type of OS preparation, and dose (Supplementary Figure S5, Supplementary Table S4).

**FT4** Eight studies reported the effect of OS preparations on FT4 in HT patients with hypothyroidism (Aisha, 2016; He et al., 2016; Yang et al., 2018a; Yang et al., 2018b; Kang and Piao, 2019; Bai et al., 2020; Jin and Wang, 2020; Zhan and Chen, 2021). Random effects models were used to analyze pooled data due to the high heterogeneity among studies (*p* < 0.00001, I^2^ = 94%). Meta-analysis showed that the experimental group was more significant than the control group in elevating the FT4 level [SMD = 1.34, 95% CI (0.59, 2.08), *p* = 0.0004] (Figure 5B). Subgroup analyses could not explain the heterogeneity (Supplementary Figure S6, Supplementary Table S4).

**TSH** Nine studies reported the effect of OS preparations on TSH in HT patients with hypothyroidism (Aisha, 2016; He et al., 2016; Yang et al., 2018a; Yang et al., 2018b; Kang and Piao, 2019; Bai et al., 2020; Jin and Wang, 2020; Wang et al., 2021; Zhan and Chen, 2021). Heterogeneity analysis showed significant heterogeneity among the studies (*p* < 0.00001, I^2^ = 96%). Therefore, meta-analysis was performed using random effects models. The results showed that there was no statistically significant difference between the test group and the control group in reducing TSH [SMD = −0.80, 95% CI (−1.71, 0.11), *p* = 0.08] (Figure 5C). There was still heterogeneity in subgroups of intervention duration and dose of OS preparation. Heterogeneity was significantly decreased in the Jinshuibao capsule subgroup, which suggests that the type of OS preparation may be the source of heterogeneity (Supplementary Figure S7, Supplementary Table S4).

#### 3.4.2 Secondary outcomes

**FIGURE 6:**
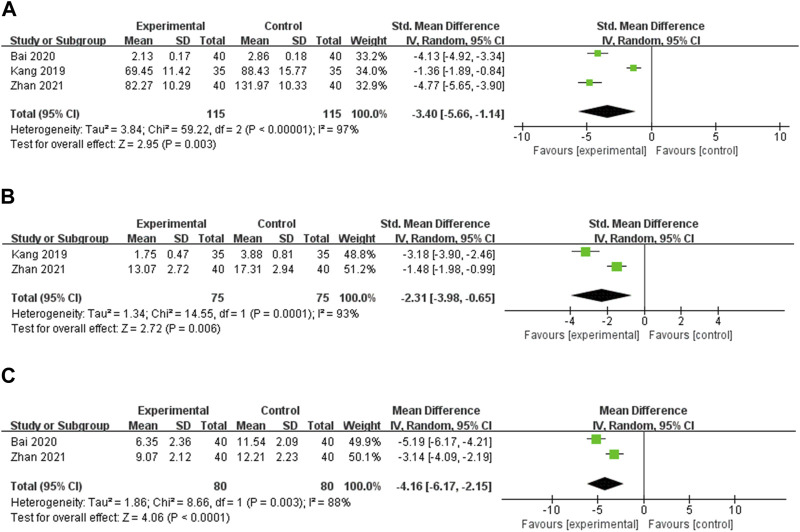
Meta-analysis results for the effect of OS preparations on inflammatory cytokines in HT patients. **(A)** TNF-α; **(B)** IL-2; **(C)** IL-6.

**TNF-α** TNF-α was reported in 3 studies (Kang and Piao, 2019; Bai et al., 2020; Zhan and Chen, 2021). Random effects models were used to analyze the pooled data [SMD = −3.40, 95% CI (−5.66, −1.14), *p* = 0.003] due to significant heterogeneity (*p* < 0.00001, I^2^ = 97%). Meta-analysis showed that OS preparation combined with LT4 significantly reduced TNF-α compared with LT4 (Figure 6A). Subgroup analyses indicated significantly decreased heterogeneity in the group with intervention durations of less than 24 weeks. This suggests that intervention duration may be a source of heterogeneity (Supplementary Figure S8, Supplementary Table S4).

**IL-2** IL-2 was reported in two studies (Kang and Piao, 2019; Zhan and Chen, 2021). Random effects models were used to analyze the combined data [SMD = −2.31, 95% CI (−3.98, −0.65), *p* = 0.006] due to significant heterogeneity (*p* < 0.0001, I^2^ = 93%). Meta-analysis showed that OS preparation combined with LT4 significantly reduced IL-2 compared with LT4 (Figure 6B).

**IL-6** IL-6 was reported in 2 studies (Bai et al., 2020; Zhan and Chen, 2021). Heterogeneity analysis showed statistical heterogeneity among the studies (*p* = 0.003, I^2^ = 88%), so meta-analysis was performed using random effects models. The results showed that OS preparations combined with LT4 reduced IL-6 more significantly than LT4 [MD = −4.16, 95% CI (−6.17, −2.15), *p* < 0.0001] (Figure 6C).

#### 3.4.3 Additional outcomes

Five studies reported adverse events (Yang et al., 2018a; Yang et al., 2018b; Kang and Piao, 2019; Bai et al., 2020; Zhan and Chen, 2021). The total sample size was 375, including 190 patients in the experimental group and 185 patients in the control group. One study reported that three patients developed symptoms of pharyngeal discomfort, and one patient developed pruritus (Yang et al., 2018a). However, the study did not provide sufficient information on the occurrence of adverse reactions in patients in the experimental and control groups. Three studies reported a total of 10 patients with adverse reactions in the experimental group, including gastrointestinal reactions in six patients, nausea in two patients, insomnia in 1 patient, and palpitations in one patient. Adverse reactions were also observed in 10 patients in the control group, respectively: gastrointestinal discomfort in two patients, nausea and vomiting in 4 patients, insomnia in one patient, palpitations in two patients, and fever in one patient (Yang et al., 2018b; Bai et al., 2020; Zhan and Chen, 2021). One study reported no adverse reactions in either group during the trial (Kang and Piao, 2019). Details of adverse events are shown in the Supplementary Table S5.

#### 3.4.4 Subgroup analysis

The effects of OS on thyroid autoantibodies, thyroid function, and inflammatory cytokines in patients with HT were investigated by subgroup analyses of different intervention durations (<24 weeks and ≥24 weeks), different OS preparation types (Bailin capsules and Jinshui Bao capsules), and different doses (≤3 g/d and >3 g/d). Subgroup analyses showed that FT3 was elevated by either the coadministration of Bailing capsule [SMD = 1.29, 95% CI (0.44, 2.13), *p* = 0.003] or the coadministration of OS preparations >3 g/d [SMD = 1.42, 95% CI (0.39, 2.45), *p* = 0.007], compared with LT4 treatment. Compared with the control group, the OS combined with LT4 intervention for ≥24 weeks group [SMD = 0.55, 95% CI (−0.12, 1.23), *p* = 0.11] and the Jinshuibao capsule combined with LT4 group [SMD = 2.28, 95% CI (−1.17, 5.73), *p* = 0.19] did not improve FT4 in patients with HT combined with hypothyroidism. Jinshuibao capsule combined with LT4 could reduce TSH in patients with HT combined with hypothyroidism [SMD = −2.48, 95% CI (−2.91, −2.05), *p* < 0.0001]. Details of the subgroup analyses are shown in Supplementary Figure S1–S8 and Supplementary Table S4.

#### 3.4.5 Sensitivity analysis

Sensitivity analysis showed that the results were relatively reliable for outcomes other than FT3, TSH, and TNF-α. In the sensitivity analysis of FT3, the meta-analysis result changed from [SMD = 0.83, 95% CI (−0.12, 1.78), *p* = 0.09] to [SMD = 1.22, 95% CI (0.51, 1.93), *p* = 0.0008] after removing the trial (Bai et al., 2020). In the sensitivity analysis of TSH, the meta-analysis result changed from [SMD = −0.80, 95% CI (−1.71, 0.11), *p* = 0.08] to [SMD = −1.08, 95% CI (−1.91, −0.25), *p* = 0.01] after removing the trial (Zhan and Chen, 2021). In the sensitivity analysis of TNF-α, the meta-analysis result changed from [SMD = −3.40, 95% CI (−5.66, −1.14), *p* = 0.003] to [SMD = −3.05, 95% CI (−6.39, 0.29), *p* = 0.07] after removing the trial (Bai et al., 2020). Supplementary Table S6 contains all of the results of the sensitivity analysis.

#### 3.4.6 Publication bias assessment

Since the number of trials included in the meta-analysis in this study was all less than 10, the Egger’s test was not used to assess publication bias. In this study, we systematically and comprehensively searched Chinese and English databases to minimize the possibility of missed detection. However, some unpublished studies with negative results could not be excluded.

#### 3.4.7 GRADE evidence rating

We used the GRADE system to assess the overall evidence for the nine outcomes described above. The quality of evidence for all outcomes was very low (Supplementary Table S7). The downgrading of evidence was mainly due to serious methodological problems in the original studies, significant heterogeneity, and suspected publication bias.

## 4 Discussion

### 4.1 Main findings

HT is a common organ-specific autoimmune disease. The presence of lymphocytic infiltration and follicular destruction in the thyroid tissue of HT patients can lead to thyroid atrophy or fibrosis. Significantly elevated serum TPOAb and TgAb titers are one of the hallmarks of the disease. Especially before the onset of hypothyroidism, antibody positivity is the only basis for the diagnosis of HT. Previous studies have found that TPOAb and TgAb levels are significantly and negatively correlated with quality of life in HT patients (Djurovic et al., 2018). Huber et al. (2002) found that positive microsomal antibodies and impaired thyroid reserve increased the risk of overt hypothyroidism in patients with subclinical hypothyroidism (TSH >6 mU/L). Similarly, in patients with autoimmune thyroiditis, substantially elevated TPOAb levels (>500 IU/mL) were associated with a modestly increased risk of hypothyroidism (Ehlers et al., 2016). In addition, much high-quality evidence suggests that the presence of TPOAb, even in the absence of thyroid dysfunction, can lead to adverse maternal and fetal outcomes in pregnancy, particularly miscarriage and preterm birth (Dhillon-Smith and Coomarasamy, 2020). Although some drugs have been shown to reduce TPOAb and TgAb levels in patients with HT, their clinical use remains limited (Toulis et al., 2010; Wichman et al., 2016; Jia et al., 2020). Therefore, it becomes critical to seek additional interventions to treat HT and delay the onset of clinical hypothyroidism.

OS is a highly promising Chinese herb that has been used in China for centuries. Bailing capsule and Jinshuibao capsule are OS preparations in capsule form. OS has been widely used in therapeutic studies for a variety of autoimmune diseases, including experimental autoimmune encephalomyelitis, IgA nephropathy, experimental autoimmune thyroiditis (EAT), and type 1 diabetes (Wang et al., 2013; Zhong et al., 2017; Xiao et al., 2018; Yang et al., 2021). In the field of HT, He et al. (2016) demonstrated that OS preparations not only significantly reduced TPOAb levels in HT patients but also had a modulating effect on the balance between helper T cells and cytotoxic T cells. In a mouse model of EAT, the *Cordyceps sinensis*-derived fungus *Isaria felina* (600 mg/kg) significantly reduced serum TSH, TPOAb, and TgAb levels and attenuated histopathological abnormalities of the thyroid gland. Mechanistically, Isaria felina reduced thyroid cell apoptosis by upregulating Bcl-2 protein expression and inhibiting cleaved caspase-3 protein expression (Yang et al., 2021). A total of 14 RCTs involving 1,014 patients with HT were included in this systematic review and meta-analysis. The results showed that OS preparations combined with a low-iodine diet or LT4 were more advantageous in decreasing circulating TPOAb and TgAb levels compared to a low-iodine diet or LT4 treatment alone. These findings are consistent with modern clinical studies of OS preparations (Zeng et al., 2014; Zhang and Li, 2023) and the results of the meta-analysis by Liu et al. (2020). In HT patients with hypothyroidism, OS preparations in combination with LT4 were more effective in elevating FT4 than LT4 alone. However, the results of the present study showed that OS preparations combined with LT4 had no significant effect on circulating FT3 and TSH.

Thyroid peroxidase (TPO) is a key enzyme involved in thyroid hormone synthesis. Generally, TPO is located on the membrane of thyroid follicular epithelial cells. The synthesis of TPOAb by thyroid tissue can be prompted when various factors cause destruction of the cellular structure. TPOAb directly destroys thyroid follicular cells through antibody-dependent cell-mediated cytotoxicity. TPOAb can also bind to TPO, thereby inactivating TPO and leading to impaired thyroid hormone synthesis. Most of the current research on the relationship between thyroid autoantibodies and thyroid function has focused on TPOAb. Previous studies have shown a close correlation between TPOAb titer and the degree of lymphocytic infiltration within the thyroid gland and hypothyroidism (Prummel and Wiersinga, 2005; Chou et al., 2015). Ghoraishian et al. (2006) also reported that TPOAb was associated with elevated TSH and decreased T4. The current meta-analysis showed that supplementation with OS preparations may decrease TPOAb and TgAb and elevate FT4 in HT patients, but this finding needs to be demonstrated in more RCTs.

Cytokines play an important role in the pathogenesis of autoimmune thyroiditis (Ganesh et al., 2011). Serum TNF-α levels were significantly elevated in HT patients compared to the healthy population. Moreover, TNF-α levels were significantly and positively correlated with TgAb and thyroid microsomal antibodies (Xie et al., 2009). Liu et al. (2016) found that serum IL-6 and IL-10 levels were significantly higher in patients with severe HT than in patients with mild HT. Previous studies have confirmed that a variety of cytokines (e.g., IL-1α, IL-1β, IL-2, IL-4, IL-6, etc.) in thyroid follicular cells enhance the inflammatory response through the production of nitric oxide and prostaglandins (Khan et al., 2015). Mori et al. (2005) observed the autoinduction of TNF-α in rat thyroid cells. Additionally, in human thyroid cells, TNF-α has a promoting effect on the expression of several inflammasome components (Guo et al., 2018). Conversely, inhibiting TLR2-HMGB1 signaling and decreasing the production of pro-inflammatory cytokines such as TNF-α, IL-1β, and IL-6 contribute to the amelioration of autoimmune thyroiditis (Li et al., 2017). Anti-TNF-α treatment not only attenuated the fibrosis of granulomatous EAT but also promoted the regression of inflammation (Chen et al., 2007). More importantly, Yang et al. (2021) demonstrated that the OS-derived fungus *Isaria felina* reduced circulating INF-γ, TNF-α, IL-6, and IL-10 levels in EAT mice. In this study, we also investigated the effect of OS preparations on pro-inflammatory cytokines TNF-α, IL-2, and IL-6 in HT patients. The results showed that the OS preparations in combination with LT4 were more beneficial than LT4 in reducing TNF-α, IL-2, and IL-6. However, the sample sizes for these outcomes were small, with the largest TNF-α (3 trials, 230 participants) and the smallest IL-2 (2 trials, 150 participants). The high degree of heterogeneity and risk of bias among studies may affect the reliability of the results. Sensitivity analysis suggested the results were relatively stable for TPOAb, TgAb, and FT4 outcomes. There was a change in the TNF-α combined effect value, with the difference changing from statistically significant to not statistically significant. Therefore, the ameliorative effect of OS preparations on TNF-α in HT patients warrants further investigation.

### 4.2 Strengths and limitations

This is the first systematic review and meta-analysis of OS preparations for HT in English. In comparison to the study published by Liu et al. (2020)., the present study included other OS preparations except for the Bailing capsule. In the included studies, we added newly published RCTs from January 2019 to June 2023. In terms of outcomes, in addition to thyroid autoantibodies, inflammatory cytokines were also investigated in this study. In addition, thyroid function (FT3, FT4, and TSH) in patients with hypothyroidism was also a new outcome added to this study. For statistical analysis, we not only used sensitivity analysis to determine the stability of the meta-analysis results but also incorporated the GRADE system methodology for a sufficient assessment of the quality of the combined body of evidence. However, there are still some limitations to this study. First, the implementation sites of the included studies were all in mainland China, so the results of this study are only informative for HT patients in this region and cannot yet be extrapolated to other countries. Second, the minimum sample of included studies was 47 cases, and the maximum sample was 120 cases. These studies were small-sample, single-center RCTs. Furthermore, none of the studies reported a basis for sample size measurement. Third, the methodological quality of the included studies was generally low. About 50% of the trials did not describe a specific randomization method. Only one trial mentioned blinding. All trials did not report allocation concealment, registration of study protocols, conflicts of interest, etc. Potential selective bias and confounding bias during trials may reduce the internal veracity of study results. Fourth, we did not use the Egger’s test to assess publication bias due to the inclusion of fewer than 10 studies in the meta-analysis. Therefore, publication bias may exist in the results of the current study. Fifth, only five studies reported the occurrence of adverse reactions and lacked long-term follow-up information, so it is not yet possible to accurately evaluate the safety of OS preparations. Sixth, the longest treatment duration in the included studies was 28 weeks, while the shortest was only 8 weeks, and there was a lack of long-term follow-up after treatment. Since HT is a chronic disease, it is unclear whether OS preparations would have a persistent effect following treatment withdrawal. Seventh, limited by the total number of included studies, some of the outcomes of this meta-analysis had only 1 included study in certain subgroups when subgroup analysis were done (e.g., Supplementary Figures S1, S3, S8). The results of subgroup analyses may be affected by this low-quality study and become unstable or unreliable. Therefore, conclusions from this portion of the subgroup analysis need to be treated with caution. Finally, Due to the language limitations of the researchers, we only searched the Chinese and English databases.

### 4.3 Implication

HT has been a therapeutic challenge in the thyroid field since it was first reported by the Japanese scholar Hashimoto in 1912. Patients are advised to have a low-iodine diet. Use LT4 to maintain the patient’s thyroid function index within the normal range during the hypothyroidism period. These are the main clinical treatment measures at present. However, the therapeutic effect of these measures is very limited. Moreover, the effect of long-term treatment with LT4 on patients’ cognitive function remains controversial. Kramer et al. (2009) found that long-term treatment with LT4 was not associated with impaired cognitive function in elderly patients. In contrast, Djurovic et al. (2018) observed that HT patients on long-term LT4 replacement therapy showed persistent impairment in both cognitive function and general wellbeing. The present study was conducted through a systematic review and meta-analysis of OS preparations (Bailin capsule and Jinshuibao capsule). We found that compared with low iodine diet or LT4, the combined intervention of OS preparations was more effective in reducing the levels of thyroid autoantibodies and pro-inflammatory cytokines in HT patients. Moreover, the OS preparation also improved FT4 in patients with HT combined with hypothyroidism. These pieces of evidence provide some references for clinical treatment. However, due to the low methodological quality of the studies included in this systematic review, the internal reality of the evidence is poor, and the safety of drug application is not clear. Therefore, clinicians should consider the actual situation of the patients before applying them rationally. Meanwhile, it is recommended that future investigators refer to the standard specifications for clinical trials to design and conduct high-quality, standardized RCTs to further improve the methodological and reporting quality of clinical trials.

## 5 Conclusion

In conclusion, the combination of OS preparations on the basis of a low-iodine diet or LT4 therapy may have potential advantages in reducing TPOAb, TgAb, TNF-α, IL-2, and IL-6 levels in HT patients. In hypothyroid HT patients, OS preparations in combination with LT4 are more beneficial than LT4 in elevating FT4. However, this systematic review was limited by the number and methodological quality of the included studies. Therefore, the current conclusions require further validation with large samples, multicenters, and high-quality RCTs.

## Data Availability

The original contributions presented in the study are included in the article/Supplementary Material, further inquiries can be directed to the corresponding author.
