# Supplementary material for: Efficacy and safety of Ophiocordyceps sinensis in the treatment of Hashimoto’s thyroiditis: a systematic review and meta-analysis
Source: Front Pharmacol. 2023 Oct 3;14:1272124. doi: 10.3389/fphar.2023.1272124 (PMC10579621; doi:10.3389/fphar.2023.1272124)

**Efficacy and safety of *Ophiocordyceps sinensis* in the treatment of Hashimoto's thyroiditis: A systematic review and a meta-analysis**

**Supplementary Files**

[Supplementary Table 1: PRISMA 2020 Checklist 1](#_Toc141693009)

[Supplementary Table 2 Systematic search detail (record number: 2023.6.7) 10](#_Toc141693010)

[Supplementary Table 3 Details of the](#_Toc141693011) *[Ophiocordyceps sinensis](#_Toc141693011)* [preparations used in the included studies 13](#_Toc141693011)

[Supplementary Table 4 Subgroup analysis of FT3, FT4, TSH, TPOAb, TgAb, and TNF-α 14](#_Toc141693012)

[Supplementary Table 5 Details of adverse reactions 16](#_Toc141693013)

[Supplementary Table 6 Sensitivity analysis 17](#_Toc141693014)

[Supplementary Tabel 7 GRADE Summary of Outcomes for](#_Toc141693015) *[Ophiocordyceps sinensis](#_Toc141693015)* [Combined with LID/LT4 compared to LID/LT4 for HT 19](#_Toc141693015)

[Supplementary Fig. 1 Subgroup analysis of TPOAb (HT patients with normal thyroid function) 21](#_Toc141693016)

[Supplementary Fig. 2 Subgroup analysis of TPOAb (HT patients with hypothyroidism) 22](#_Toc141693017)

[Supplementary Fig. 3 Subgroup analysis of TgAb (HT patients with normal thyroid function) 23](#_Toc141693018)

[Supplementary Fig. 4 Subgroup analysis of TgAb (HT patients with hypothyroidism) 24](#_Toc141693019)

[Supplementary Fig. 5 Subgroup analysis of FT3 (HT patients with hypothyroidism) 25](#_Toc141693020)

[Supplementary Fig. 6 Subgroup analysis of FT4 (HT patients with hypothyroidism) 26](#_Toc141693021)

[Supplementary Fig. 7 Subgroup analysis of TSH (HT patients with hypothyroidism) 27](#_Toc141693022)

[Supplementary Fig. 8 Subgroup analysis of TNF-α 28](#_Toc141693023)

# Supplementary Table 1: PRISMA 2020 Checklist

| **Section and Topic** | **Item #** | **Checklist item** | **Location where item is reported** |
| --- | --- | --- | --- |
| **TITLE** | | |  |
| Title | 1 | Identify the report as a systematic review. | Page 1, Line 1-2. |
| **ABSTRACT** | | |  |
| Abstract | 2 | See the PRISMA 2020 for Abstracts checklist. | Page 1-2, Line 8-38. |
| **INTRODUCTION** | | |  |
| Rationale | 3 | Describe the rationale for the review in the context of existing knowledge. | Page 2-3, Line 44-79. |
| Objectives | 4 | Provide an explicit statement of the objective(s) or question(s) the review addresses. | Page 3, Line 79-83. |
| **METHODS** | | |  |
| Eligibility criteria | 5 | Specify the inclusion and exclusion criteria for the review and how studies were grouped for the syntheses. | Page 4, Line 92-116. |
| Information sources | 6 | Specify all databases, registers, websites, organisations, reference lists and other sources searched or consulted to identify studies. Specify the date when each source was last searched or consulted. | Page 5, Line 118-121. |
| Search strategy | 7 | Present the full search strategies for all databases, registers and websites, including any filters and limits used. | Page 5  Supplementary Table 2 |
| Selection process | 8 | Specify the methods used to decide whether a study met the inclusion criteria of the review, including how many reviewers screened each record and each report retrieved, whether they worked independently, and if applicable, details of automation tools used in the process. | Page 5, Line 129-133, 139-140. |
| Data collection process | 9 | Specify the methods used to collect data from reports, including how many reviewers collected data from each report, whether they worked independently, any processes for obtaining or confirming data from study investigators, and if applicable, details of automation tools used in the process. | Page 5, Line 134-140. |
| Data items | 10a | List and define all outcomes for which data were sought. Specify whether all results that were compatible with each outcome domain in each study were sought (e.g. for all measures, time points, analyses), and if not, the methods used to decide which results to collect. | Page 5, Line 134-139. |
|  | 10b | List and define all other variables for which data were sought (e.g. participant and intervention characteristics, funding sources). Describe any assumptions made about any missing or unclear information. | Page 5, Line 134-139. |
| Study risk of bias assessment | 11 | Specify the methods used to assess risk of bias in the included studies, including details of the tool(s) used, how many reviewers assessed each study and whether they worked independently, and if applicable, details of automation tools used in the process. | Page 5-6, Line 142-148. |
| Effect measures | 12 | Specify for each outcome the effect measure(s) (e.g. risk ratio, mean difference) used in the synthesis or presentation of results. | Page 6, Line 152-156. |
| Synthesis methods | 13a | Describe the processes used to decide which studies were eligible for each synthesis (e.g. tabulating the study intervention characteristics and comparing against the planned groups for each synthesis (item #5)). | Page 6, Line 151-164. |
|  | 13b | Describe any methods used to tabulate or visually display results of individual studies and syntheses. | Page 6, Line 151-164. |
|  | 13c | Describe any methods used to tabulate or visually display results of individual studies and syntheses. | Page 6, Line 150-173. |
|  | 13d | Describe any methods used to synthesize results and provide a rationale for the choice(s). If meta-analysis was performed, describe the model(s), method(s) to identify the presence and extent of statistical heterogeneity, and software package(s) used. | Page 6, Line 150-151, 159-164. |
|  | 13e | Describe any methods used to explore possible causes of heterogeneity among study results (e.g. subgroup analysis, meta-regression). | Page 6, Line 165-169. |
|  | 13f | Describe any sensitivity analyses conducted to assess robustness of the synthesized results. | Page 6, Line 169-170. |
| Reporting bias assessment | 14 | Describe any methods used to assess risk of bias due to missing results in a synthesis (arising from reporting biases). | Page 6, Line 170-173. |
| Certainty assessment | 15 | Describe any methods used to assess certainty (or confidence) in the body of evidence for an outcome. | Page 7, Line 175-179. |
| **RESULTS** | | |  |
| Study selection | 16a | Describe the results of the search and selection process, from the number of records identified in the search to the number of studies included in the review, ideally using a flow diagram. | Page 7, Line 182-193. Figure 1 |
|  | 16b | Cite studies that might appear to meet the inclusion criteria, but which were excluded, and explain why they were excluded. | Page 7, Line 185-191. |
| Study characteristics | 17 | Cite each included study and present its characteristics. | Page 7-8, Line 195-213.  Table 1 |
| Risk of bias in studies | 18 | Present assessments of risk of bias for each included study. | Page 8, Line 215-230.  Figure 2 |
| Results of individual studies | 19 | For all outcomes, present, for each study: (a) summary statistics for each group (where appropriate) and (b) an effect estimate and its precision (e.g. confidence/credible interval), ideally using structured tables or plots. | Figure 3-6. |
| Results of syntheses | 20a | For each synthesis, briefly summarize the characteristics and risk of bias among contributing studies. | Page 8-12, Line 232-335. |
|  | 20b | Present results of all statistical syntheses conducted. If meta-analysis was done, present for each the summary estimate and its precision (e.g. confidence/credible interval) and measures of statistical heterogeneity. If comparing groups, describe the direction of the effect. | Page 8-12, Line 232-335.  Figure 3-6 |
|  | 20c | Present results of all investigations of possible causes of heterogeneity among study results. | Page 13, Line 353-367.  Supplementary Figure 1-8.  Supplementary Table 4. |
|  | 20d | Present results of all sensitivity analyses conducted to assess the robustness of the synthesized results. | Page 13-14, Line 369-379.  Supplementary Table 6 |
| Reporting biases | 21 | Present assessments of risk of bias due to missing results (arising from reporting biases) for each synthesis assessed. | Page 14, Line 381-385. |
| Certainty of evidence | 22 | Present assessments of certainty (or confidence) in the body of evidence for each outcome assessed. | Page 14, Line 387-391.  Supplementary Table 7 |
| **DISCUSSION** | | |  |
| Discussion | 23a | Provide a general interpretation of the results in the context of other evidence. | Page 14-16, Line 422-433, 451-455. |
|  | 23b | Discuss any limitations of the evidence included in the review. | Page 17, Line 475-485. |
|  | 23c | Discuss any limitations of the review processes used. | Page 17, Line 485-491. |
|  | 23d | Discuss implications of the results for practice, policy, and future research. | Page 17-18, Line 493-512. |
| **OTHER INFORMATION** | | |  |
| Registration and protocol | 24a | Provide registration information for the review, including register name and registration number, or state that the review was not registered. | Page 2, Line 39-40. |
|  | 24b | Indicate where the review protocol can be accessed, or state that a protocol was not prepared. | Page 2, Line 39-40. |
|  | 24c | Describe and explain any amendments to information provided at registration or in the protocol. | Page 2, Line 39-40. |
| Support | 25 | Describe sources of financial or non-financial support for the review, and the role of the funders or sponsors in the review. | Page 19, Line 537-539. |
| Competing interests | 26 | Declare any competing interests of review authors. | Page 19, Line 534-535. |
| Availability of data, code and other materials | 27 | Report which of the following are publicly available and where they can be found: template data collection forms; data extracted from included studies; data used for all analyses; analytic code; any other materials used in the review. | Page 19, Line 525-527. |

# Supplementary Table 2 Systematic search detail (record number: 2023.6.7)

| **The search strategy for PubMed** | | |
| --- | --- | --- |
| **Sequence** | **Search terms** | **Hits** |
| #1 | ((((((((((((((((((((((Hashimoto Disease[MeSH Terms]) OR (Disease, Hashimoto[Title/Abstract])) OR (Hashimoto Struma[Title/Abstract])) OR (Hashimoto Thyroiditis[Title/Abstract])) OR (Hashimoto Thyroiditides[Title/Abstract])) OR (Thyroiditides, Hashimoto[Title/Abstract])) OR (Thyroiditis, Hashimoto[Title/Abstract])) OR (Hashimoto's Syndrome[Title/Abstract])) OR (Hashimoto Syndrome[Title/Abstract])) OR (Hashimoto's Syndromes[Title/Abstract])) OR (Hashimotos Syndrome[Title/Abstract])) OR (Syndrome, Hashimoto's[Title/Abstract])) OR (Syndromes, Hashimoto's[Title/Abstract])) OR (Hashimoto's Struma[Title/Abstract])) OR (Chronic Lymphocytic Thyroiditis[Title/Abstract])) OR (Chronic Lymphocytic Thyroiditides[Title/Abstract])) OR (Lymphocytic Thyroiditides, Chronic[Title/Abstract])) OR (Lymphocytic Thyroiditis, Chronic[Title/Abstract])) OR (Thyroiditides, Chronic Lymphocytic[Title/Abstract])) OR (Thyroiditis, Chronic Lymphocytic[Title/Abstract])) OR (Hashimoto's Disease[Title/Abstract])) OR (Disease, Hashimoto's[Title/Abstract])) OR (Hashimotos Disease[Title/Abstract]) | 12,601 |
| #2 | ((((((((Cordyceps[MeSH Terms]) OR (Ophiocordyceps sinensis[Title/Abstract])) OR (Sphaeria sinensis[Title/Abstract])) OR (Caterpillar Fungus[Title/Abstract])) OR (Fungus, Caterpillar[Title/Abstract])) OR (Cordyceps sinensis[Title/Abstract])) OR (Cordyceps militaris[Title/Abstract])) OR (Bailing[Title/Abstract])) OR (Jinshuibao[Title/Abstract]) | 2,074 |
| #3 | #1 AND #2 | 1 |
| **The search strategy for Web of science** | | |
| **Sequence** | **Search terms** | **Hits** |
| #1 | TS=(Hashimoto Disease) | 15,991 |
| #2 | **(((((((((((((((((((((AB=(Disease, Hashimoto)) OR AB=(Hashimoto Struma)) OR AB=(Hashimoto Thyroiditis)) OR AB=(Hashimoto Thyroiditides)) OR AB=(Thyroiditides, Hashimoto)) OR AB=(Thyroiditis, Hashimoto)) OR AB=(Hashimoto's Syndrome)) OR AB=(Hashimoto Syndrome)) OR AB=(Hashimoto's Syndromes)) OR AB=(Hashimotos Syndrome)) OR AB=(Syndrome, Hashimoto's)) OR AB=(Syndromes, Hashimoto's)) OR AB=(Hashimoto's Struma)) OR AB=(Chronic Lymphocytic Thyroiditis)) OR AB=(Chronic Lymphocytic Thyroiditides)) OR AB=(Lymphocytic Thyroiditides, Chronic)) OR AB=(Lymphocytic Thyroiditis, Chronic)) OR AB=(Thyroiditides, Chronic Lymphocytic)) OR AB=(Thyroiditis, Chronic Lymphocytic)) OR AB=(Hashimoto's Disease)) OR AB=(Disease, Hashimoto's)) OR AB=(Hashimotos Disease)** | 13,679 |
| #3 | #1 OR #2 | 17,680 |
| #4 | **TS=(Cordyceps)** | 18,358 |
| #5 | **(((((((AB=(Ophiocordyceps sinensis)) OR AB=(Sphaeria sinensis)) OR AB=(Caterpillar Fungus)) OR AB=(Fungus, Caterpillar)) OR AB=(Cordyceps sinensis)) OR AB=(Cordyceps militaris)) OR AB=(Bailing)) OR AB=(Jinshuibao)** | 21,098 |
| #6 | #4 OR #5 | 26,801 |
| #7 | #3 AND #6 | 3 |
| **The search strategy for Cochrane Library** | | |
| **Sequence** | **Search terms** | **Hits** |
| #1 | MeSH descriptor:[Diabetic Nephropathies] explode all trees | 1,475 |
| #2 | (Disease, Hashimoto):ti,ab,kw OR (Hashimoto Struma):ti,ab,kw OR (Hashimoto Thyroiditis):ti,ab,kw OR (Hashimoto Thyroiditides):ti,ab,kw OR (Thyroiditides, Hashimoto):ti,ab,kw | 274 |
| #3 | (Thyroiditis, Hashimoto):ti,ab,kw OR (Hashimoto's Syndrome):ti,ab,kw OR (Hashimoto Syndrome):ti,ab,kw OR (Hashimoto's Syndromes):ti,ab,kw OR (Hashimotos Syndrome):ti,ab,kw | 249 |
| #4 | (Syndrome, Hashimoto's):ti,ab,kw OR (Syndromes, Hashimoto's):ti,ab,kw OR (Hashimoto's Struma):ti,ab,kw OR (Chronic Lymphocytic Thyroiditis):ti,ab,kw OR (Chronic Lymphocytic Thyroiditides):ti,ab,kw | 79 |
| #5 | (Lymphocytic Thyroiditides, Chronic):ti,ab,kw OR (Lymphocytic Thyroiditis, Chronic):ti,ab,kw OR (Thyroiditides, Chronic Lymphocytic):ti,ab,kw OR (Thyroiditis, Chronic Lymphocytic):ti,ab,kw OR (Hashimoto's Disease):ti,ab,kw | 272 |
| #6 | (Disease, Hashimoto's):ti,ab,kw OR (Hashimotos Disease):ti,ab,kw | 228 |
| #7 | #2 OR #3 OR #4 OR #5 OR #6 | 312 |
| #8 | #1 OR #7 | 312 |
| #9 | MeSH descriptor: [Cordyceps] explode all trees | 29 |
| #10 | (Ophiocordyceps sinensis):ti,ab,kw OR (Sphaeria sinensis):ti,ab,kw OR (Caterpillar Fungus):ti,ab,kw OR (Fungus, Caterpillar):ti,ab,kw OR (Cordyceps sinensis):ti,ab,kw OR (Cordyceps militaris):ti,ab,kw | 74 |
| #11 | #9 OR #10 | 84 |
| #12 | #8 AND #11 | 0 |
| **The search strategy for EMBASE** | | |
| **Sequence** | **Search terms** | **Hits** |
| #1 | 'hashimoto disease'/exp | 16,406 |
| #2 | 'Disease, Hashimoto':ab,ti OR 'Hashimoto Struma':ab,ti OR 'Hashimoto Thyroiditis':ab,ti OR 'Hashimoto Thyroiditides':ab,ti OR 'Thyroiditides, Hashimoto':ab,ti OR 'Thyroiditis, Hashimoto':ab,ti OR 'Hashimoto Syndrome':ab,ti OR 'Hashimotos Syndrome':ab,ti OR 'Hashimoto's Struma':ab,ti OR 'Chronic Lymphocytic Thyroiditis':ab,ti OR 'Chronic Lymphocytic Thyroiditides':ab,ti OR 'Lymphocytic Thyroiditides, Chronic':ab,ti OR 'Lymphocytic Thyroiditis, Chronic':ab,ti OR 'Thyroiditides, Chronic Lymphocytic':ab,ti OR 'Thyroiditis, Chronic Lymphocytic':ab,ti OR 'Hashimotos Disease':ab,ti | 2,990 |
| #3 | #1 OR #2 | 16,972 |
| #4 | 'Cordyceps'/exp | 1,675 |
| #5 | 'Ophiocordyceps sinensis':ab,ti OR 'Sphaeria sinensis':ab,ti OR 'Caterpillar Fungus':ab,ti OR 'Fungus, Caterpillar':ab,ti OR 'Cordyceps sinensis':ab,ti OR 'Cordyceps militaris':ab,ti | 1,810 |
| #6 | #4 OR #5 | 2,597 |
| #7 | #3 AND #6 | 1 |
| **The search strategy for CNKI** | | |
| **Sequence** | **Search terms** | **Hits** |
| #1 | (SU%=Hashimoto's thyroiditis) OR (SU%=Hashimoto thyroiditis) OR (SU%=Chronic lymphocytic thyroiditis) OR (SU%=Autoimmune thyroiditis) OR (SU%=Hashimoto's disease) OR (SU%=Hashimoto disease) | 6,895 |
| #2 | (SU%=Ophiocordyceps sinensis) OR (SU%=Bailing) OR (SU%=Jinshuibao) | - |
| #3 | #2 is retrieved in the result of #1 | 50 |
| **The search strategy for Wanfang Data** | | |
| **Sequence** | **Search terms** | **Hits** |
| #1 | (Theme=Hashimoto's thyroiditis) OR (Theme=Hashimoto thyroiditis) OR (Theme=Chronic lymphocytic thyroiditis) OR (Theme=Autoimmune thyroiditis) OR (Theme=Hashimoto's disease) OR (Theme=Hashimoto disease) | 8,225 |
| #2 | (Theme=Ophiocordyceps sinensis) OR (Theme=Bailing) OR (Theme=Jinshuibao) | - |
| #3 | #2 is retrieved in the result of #1 | 34 |
| **The search strategy for SinoMed** | | |
| **Sequence** | **Search terms** | **Hits** |
| #1 | " Hashimoto's thyroiditis "[Common fields: Auto] OR " Hashimoto thyroiditis "[Common fields: Auto] OR " Chronic lymphocytic thyroiditis "[Common fields: Auto] OR " Autoimmune thyroiditis "[Common fields: Auto] OR " Hashimoto's disease "[Common fields: Auto] OR " Hashimoto disease "[Common fields: Auto] | 7,076 |
| #2 | " Ophiocordyceps sinensis "[Common fields: Auto] OR " Bailing "[Common fields: Auto] OR " Jinshuibao "[Common fields: Auto] | 6,855 |
| #3 | #1 AND #2 | 39 |
| The common fields consist of four search terms: Chinese title, abstract, keywords, and theme words. | | |
| **The search strategy for VIP** | | |
| **Sequence** | **Search terms** | **Hits** |
| #1 | M=Hashimoto's thyroiditis+Hashimoto thyroiditis+Chronic lymphocytic thyroiditis+Autoimmune thyroiditis+Hashimoto's disease+Hashimoto disease | 5,399 |
| #2 | M=Ophiocordyceps sinensis*+*Bailing+Jinshuibao | 6,342 |
| #3 | #1 AND #2 | 32 |

# Supplementary Table 3 Details of the *Ophiocordyceps sinensis* preparations used in the included studies

| Preparation | Dosage Form | Specification | Ingredients | Approval Year | Approval Number | Manufacturer | Administ-ration | Dose | Insurance Coverage | Adverse Reactions | Contraindication |
| --- | --- | --- | --- | --- | --- | --- | --- | --- | --- | --- | --- |
| Bailing Capsule | Capsule | 0.2g/0.5g | Fermented Cordyceps sinensis powder [Cs-C-Q80] | 1988 | National medicine permission number  Z10910036 | Hangzhou Zhongmei Huadong Pharmaceutical Co., Ltd. | Oral | 1.0-3.0g/  time;  3 times/ day | National Medical Insurance (2022) | Some  patients have pharyngeal discomfort. | Unclear |
| Jinshuibao Capsule | Capsule | 0.33g | Fermented Cordyceps Fungus Powder [Cs-4] | 1987 | National medicine permission number  Z10890003 | Jiangxi Jimin Trusted Jinshuibao Pharmaceutical Co., Ltd. | Oral | 0.99-1.98g/time;  3 times/ day | National Medical Insurance (2022) | Some patients have mild gastrointestinal discomfort. Like nausea, etc. | Prohibited for those who are allergic to this product. People with allergic constitutions should use it with caution. |

# Supplementary Table 4 Subgroup analysis of TPOAb, TgAb, FT3, FT4, TSH and TNF-α

| Outcomes | Hypothyroidism | Subgroup | n | SMD/MD (95%CI) | *I^2^*(%) | *^1^P* | *^2^P* | *^3^P* |
| --- | --- | --- | --- | --- | --- | --- | --- | --- |
| TPOAb | No | Total | 4 | -3.81 [-5.07, -2.54] | 89 | <0.00001 | <0.00001 | - |
|  |  | Intervention duration  < 24week | 1 | -3.86 [-4.54, -3.17] | - | <0.00001 | - | 0.97 |
|  |  | Intervention duration  ≥ 24week | 3 | -3.82 [-5.71, -1.93] | 92 | <0.0001 | <0.00001 |  |
|  |  | Bailing Capsule | 3 | -3.90 [-5.62, -2.17] | 93 | <0.00001 | <0.00001 | 0.76 |
|  |  | Jinshuibao Capsule | 1 | -3.60 [-4.55, -2.64] | - | <0.00001 | - |  |
|  |  | Dose ≤ 3g/d | 2 | -2.90 [-4.18, -1.61] | 79 | <0.0001 | 0.03 | 0.11 |
|  |  | Dose > 3g/d | 2 | -4.73 [-6.56, -2.90] | 86 | <0.00001 | 0.007 |  |
|  | Yes | Total | 9 | -2.04 [-2.82, -1.26] | 94 | <0.00001 | <0.00001 | - |
|  |  | Intervention duration  < 24week | 5 | -2.93 [-3.59, -2.27] | 81 | <0.00001 | 0.0004 | <0.0001 |
|  |  | Intervention duration  ≥ 24week | 4 | -0.92 [-1.57, -0.27] | 84 | 0.006 | 0.0003 |  |
|  |  | Bailing Capsule | 7 | -2.13 [-3.17, -1.09] | 95 | <0.0001 | <0.00001 | 0.53 |
|  |  | Jinshuibao Capsule | 2 | -1.78 [-2.16, -1.39] | 0 | <0.00001 | 0.32 |  |
|  |  | Dose ≤ 3g/d | 3 | -2.05 [-2.65, -1.46] | 70 | <0.00001 | 0.04 | 1.00 |
|  |  | Dose > 3g/d | 6 | -2.05 [-3.23, -0.87] | 96 | 0.0006 | <0.00001 |  |
| TgAb | No | Total | 4 | -4.73 [-6.86, -2.61] | 96 | 0.0001 | <0.00001 | - |
|  |  | Intervention duration  < 24week | 1 | -3.92 [-4.61, -3.22] | - | <0.00001 | - | 0.47 |
|  |  | Intervention duration  ≥ 24week | 3 | -5.16 [-8.50, -1.82] | 97 | 0.002 | <0.00001 |  |
|  |  | Bailing Capsule | 3 | -5.78 [-8.45, -3.10] | 95 | <0.0001 | <0.00001 | 0.006 |
|  |  | Jinshuibao Capsule | 1 | -1.89 [-2.59, -1.19] | - | <0.00001 | - |  |
|  |  | Dose ≤ 3g/d | 2 | -2.68 [-4.26, -1.10] | 88 | 0.0009 | 0.004 | 0.19 |
|  |  | Dose > 3g/d | 2 | -7.20 [-13.77, -0.63] | 97 | 0.03 | <0.00001 |  |
|  | Yes | Total | 9 | -2.01 [-2.68, -1.33] | 92 | <0.00001 | <0.00001 | - |
|  |  | Intervention duration  < 24week | 5 | -2.62 [-3.69, -1.54] | 94 | <0.00001 | <0.00001 | 0.05 |
|  |  | Intervention duration  ≥ 24week | 4 | -1.30 [-2.08, -0.52] | 88 | 0.001 | <0.0001 |  |
|  |  | Bailing Capsule | 7 | -2.07 [-2.95, -1.19] | 94 | <0.00001 | <0.00001 | 0.69 |
|  |  | Jinshuibao Capsule | 2 | -1.85 [-2.41, -1.30] | 50 | <0.00001 | 0.16 |  |
|  |  | Dose ≤ 3g/d | 3 | -1.91 [-2.27, -1.56] | 20 | <0.00001 | 0.29 | 0.77 |
|  |  | Dose > 3g/d | 6 | -2.08 [-3.11, -1.05] | 95 | <0.0001 | <0.00001 |  |
| FT3 | Yes | Total | 8 | 0.83 [-0.12; 1.78] | 96 | 0.09 | <0.00001 | - |
|  |  | Intervention duration  < 24week | 4 | 0.71 [-1.02, 2.44] | 98 | 0.42 | <0.00001 | 0.82 |
|  |  | Intervention duration  ≥ 24week | 4 | 0.95 [-0.14, 2.03] | 94 | 0.09 | <0.00001 |  |
|  |  | Bailing Capsule | 6 | 1.29 [0.44, 2.13] | 94 | 0.003 | <0.00001 | 0.21 |
|  |  | Jinshuibao Capsule | 2 | -0.56 [-3.31, 2.19] | 98 | 0.69 | <0.00001 |  |
|  |  | Dose ≤ 3g/d | 3 | -0.15 [-1.84, 1.53] | 97 | 0.86 | <0.00001 | 0.12 |
|  |  | Dose > 3g/d | 5 | 1.42 [0.39, 2.45] | 95 | 0.007 | <0.00001 |  |
| FT4 | Yes | Total | 8 | 1.34 [0.59, 2.08] | 94 | 0.0004 | <0.00001 | - |
|  |  | Intervention duration  < 24week | 4 | 2.15 [0.95, 3.34] | 95 | 0.0004 | <0.00001 | 0.02 |
|  |  | Intervention duration  ≥ 24week | 4 | 0.55 [-0.12, 1.23] | 86 | 0.11 | <0.0001 |  |
|  |  | Bailing Capsule | 6 | 1.04 [0.38, 1.71] | 91 | 0.002 | <0.00001 | 0.049 |
|  |  | Jinshuibao Capsule | 2 | 2.28 [-1.17, 5.73] | 98 | 0.19 | <0.00001 |  |
|  |  | Dose ≤ 3g/d | 3 | 1.75 [-0.02, 3.52] | 97 | 0.05 | <0.00001 | 0.52 |
|  |  | Dose > 3g/d | 5 | 1.11 [0.28, 1.93] | 92 | 0.009 | <0.00001 |  |
| TSH | Yes | Total | 9 | -0.80 [-1.71, 0.11] | 96 | 0.08 | <0.00001 | - |
|  |  | Intervention duration  < 24week | 5 | -0.64 [-1.83, 0.56] | 97 | 0.30 | <0.00001 | 0.72 |
|  |  | Intervention duration  ≥ 24week | 4 | -1.01 [-2.65, 0.63] | 97 | 0.23 | <0.00001 |  |
|  |  | Bailing Capsule | 7 | -0.33[-1.24, 0.58] | 96 | 0.48 | <0.00001 | <0.0001 |
|  |  | Jinshuibao Capsule | 2 | -2.48 [-2.91, -2.05] | 0 | <0.00001 | 0.81 |  |
|  |  | Dose ≤ 3g/d | 3 | -1.17 [-3.88, 1.54] | 99 | 0.40 | <0.00001 | 0.70 |
|  |  | Dose > 3g/d | 6 | -0.62 [-1.46, 0.22] | 94 | 0.15 | <0.00001 |  |
| TNF-α | Yes | Total | 3 | -3.40 [-5.66, -1.14] | 97 | 0.003 | <0.00001 | - |
|  |  | Intervention duration  < 24week | 2 | -4.42 [-5.05, -3.79] | 13 | <0.00001 | 0.28 | <0.00001 |
|  |  | Intervention duration  ≥ 24week | 1 | -1.36 [-1.89, -0.84] | - | <0.00001 | - |  |
|  |  | Bailing Capsule | 1 | -4.77 [-5.65, -3.90] | - | <0.00001 | - | 0.16 |
|  |  | Jinshuibao Capsule | 2 | -2.73 [-5.44, -0.02] | 97 | 0.05 | <0.00001 |  |
|  |  | Dose ≤ 3g/d | 3 | -3.40 [-5.66, -1.14] | 97 | 0.003 | <0.00001 | - |
|  |  | Dose > 3g/d | - | - | - | - | - |  |

^1^p values for effect size; ^2^p values for heterogeneity; ^3^p values for between subgroup.

# Supplementary Table 5 Details of adverse reactions

| Study | Sample size | | Pharyngeal discomfort | | Pruritus | | Gastrointestinal reactions | | nausea or vomiting | | Insomnia | | Palpitations | | Fever | |
| --- | --- | --- | --- | --- | --- | --- | --- | --- | --- | --- | --- | --- | --- | --- | --- | --- |
|  | E | C | E | C | E | C | E | C | E | C | E | C | E | C | E | C |
| Yang et al., 2018a | 35 | 30 | 3 | | 1 | | - | - | - | - | - | - | - | - | - | - |
| Bai et al., 2020 | 40 | 40 | - | - | - | - | 4 | 2 | 2 | 2 | - | - | - | - | - | - |
| Yang et al., 2018b | 40 | 40 | - | - | - | - | 2 | - | - | 2 | - | - | - | - | - | - |
| Zhan and Chen, 2021 | 40 | 40 | - | - | - | - | - | - | - | - | 1 | 1 | 1 | 2 | - | 1 |
| Kang and Piao, 2019 | 35 | 35 | - | - | - | - | - | - | - | - | - | - | - | - | - | - |

E: Experimental group; C: control group.

# Supplementary Table 6 Sensitivity analysis

| Outcome | Hypothyroidism | Omitted Study | Data with study removed MD/SMD (95% CI) | *P* | *I^2^* |
| --- | --- | --- | --- | --- | --- |
| TPOAb | No | Li 2017 | -3.82 [-5.71, -1.93] | <0.0001 | 92% |
|  |  | Tan 2021 | -3.90 [-5.62, -2.17] | <0.00001 | 93% |
|  |  | Zhang 2015 | -4.32 [-5.45, -3.19] | <0.00001 | 78% |
|  |  | Zhu 2020 | -3.23 [-4.27, -2.19] | <0.00001 | 82% |
|  | Yes | Aisha 2016 | -1.89 [-2.70, -1.09] | <0.00001 | 94% |
|  |  | Bai 2020 | -2.06 [-2.95, -1.17] | < 0.00001 | 95% |
|  |  | He 2016 | -2.16 [-3.02, -1.29] | < 0.00001 | 95% |
|  |  | Jin 2020 | -1.95 [-2.78, -1.12] | < 0.00001 | 94% |
|  |  | Kang 2019 | -2.11 [-3.00, -1.22] | < 0.00001 | 95% |
|  |  | Wang 2021 | -1.78 [-2.53, -1.04] | < 0.00001 | 93% |
|  |  | Yang 2018a | -2.29 [-2.98, -1.60] | < 0.00001 | 91% |
|  |  | Yang 2018b | -2.19 [-3.06, -1.32] | < 0.00001 | 94% |
|  |  | Zhan 2021 | -1.97 [-2.82, -1.12] | < 0.00001 | 94% |
| TgAb | No | Li 2017 | -5.16 [-8.50, -1.82] | 0.002 | 97% |
|  |  | Tan 2021 | -5.78 [-8.45, -3.10] | <0.0001 | 95% |
|  |  | Zhang 2015 | -5.27 [-8.40, -2.15] | 0.0009 | 97% |
|  |  | Zhu 2020 | -3.10 [-4.38, -1.82] | <0.00001 | 89% |
|  | Yes | Aisha 2016 | -1.71 [-2.30, -1.12] | <0.00001 | 90% |
|  |  | Bai 2020 | -2.07 [-2.85, -1.29] | <0.00001 | 93% |
|  |  | He 2016 | -2.08 [-2.83, -1.32] | <0.00001 | 93% |
|  |  | Jin 2020 | -2.13 [-2.93, -1.34] | <0.00001 | 93% |
|  |  | Kang 2019 | -1.99 [-2.74, -1.24] | <0.00001 | 93% |
|  |  | Wang 2021 | -1.76 [-2.38, -1.14] | <0.00001 | 90% |
|  |  | Yang 2018a | -2.22 [-2.88, -1.56] | <0.00001 | 90% |
|  |  | Yang 2018b | -2.11 [-2.89, -1.33] | <0.00001 | 93% |
|  |  | Zhan 2021 | -2.01 [-2.77, -1.25] | <0.00001 | 93% |
| FT3 | Yes | Aisha 2016 | 0.58 [-0.40, 1.55] | 0.24 | 96% |
|  |  | Bai 2020 | 1.22 [0.51, 1.93] | 0.0008 | 93% |
|  |  | He 2016 | 0.91 [-0.16, 1.99] | 0.10 | 97% |
|  |  | Jin 2020 | 0.73 [-0.36, 1.81] | 0.19 | 96% |
|  |  | Kang 2019 | 0.83 [-0.28, 1.94] | 0.14 | 97% |
|  |  | Yang 2018a | 0.94 [-0.14, 2.02] | 0.09 | 97% |
|  |  | Yang 2018b | 0.57 [-0.38, 1.51] | 0.24 | 96% |
|  |  | Zhan 2021 | 0.86 [-0.27, 1.98] | 0.13 | 97% |
| FT4 | Yes | Aisha 2016 | 1.18 [0.41, 1.95] | 0.003 | 94% |
|  |  | Bai 2020 | 0.97 [0.39, 1.54] | 0.001 | 89% |
|  |  | He 2016 | 1.49 [0.67, 2.30] | 0.0003 | 94% |
|  |  | Jin 2020 | 1.32 [0.44, 2.20] | 0.003 | 95% |
|  |  | Kang 2019 | 1.46 [0.61, 2.30] | 0.0007 | 94% |
|  |  | Yang 2018a | 1.54 [0.78, 2.30] | <0.0001 | 93% |
|  |  | Yang 2018b | 1.32 [0.46, 2.17] | 0.002 | 95% |
|  |  | Zhan 2021 | 1.43 [0.56, 2.29] | 0.001 | 95% |
| TSH | Yes | Aisha 2016 | -0.80 [-1.83, 0.23] | 0.13 | 97% |
|  |  | Bai 2020 | -0.59 [-1.50, 0.33] | 0.21 | 96% |
|  |  | He 2016 | -0.92 [-1.92, 0.08] | 0.07 | 97% |
|  |  | Jin 2020 | -0.76 [-1.82, 0.31] | 0.16 | 97% |
|  |  | Kang 2019 | -0.60 [-1.54, 0.33] | 0.21 | 96% |
|  |  | Wang 2021 | -0.89 [-1.92, 0.13] | 0.09 | 97% |
|  |  | Yang 2018a | -0.98 [-1.95, -0.02] | 0.05 | 96% |
|  |  | Yang 2018b | -0.59 [-1.52, 0.33] | 0.21 | 96% |
|  |  | Zhan 2021 | -1.08 [-1.91, -0.25] | 0.01 | 95% |
| TNF-α | Yes | Bai 2020 | -3.05 [-6.39, 0.29] | 0.07 | 98% |
|  |  | Kang 2019 | -4.42 [-5.05, -3.79] | <0.00001 | 13% |
|  |  | Zhan 2021 | -2.73 [-5.44, -0.02] | 0.05 | 97% |
| IL-2 | Yes | Kang 2019 | -1.48 [-1.98, -0.99] | <0.00001 | 93% |
|  |  | Zhan 2021 | -3.18 [-3.90, -2.46] | <0.00001 | 93% |
| IL-6 | Yes | Bai 2020 | -3.14 [-4.09, -2.19] | <0.00001 | 88% |
|  |  | Zhan 2021 | -5.19 [-6.17, -4.21] | <0.00001 | 88% |

# Supplementary Tabel 7 GRADE Summary of Outcomes for *Ophiocordyceps sinensis* Combined with LID/LT4 compared to LID/LT4 for HT

| Outcomes | Hypothyroidism | Risk of bias | Inconsistency | Indirectness | Imprecision | Other  considerations | No. of patients (studies) | Relative  (95% CI) | Absolute  (95% CI) | Certainty of the evidence | Importance |
| --- | --- | --- | --- | --- | --- | --- | --- | --- | --- | --- | --- |
| TPOAb | No | serious^a^ | serious^b^ | not serious | not serious | publication bias strongly suspected^d^ | 259(4) | - | SMD3.81 lower(5.07 lower to 2.54 lower) | ⨁◯◯◯Very low | IMPORTANT |
|  | Yes | serious^a^ | serious^b^ | not serious | not serious | publication bias strongly suspected^d^ | 672(9) | - | SMD 2.04 lower(2.82 lower to 1.26 lower) | ⨁◯◯◯Very low | IMPORTANT |
| TgAb | No | serious^a^ | serious^b^ | not serious | not serious | publication bias strongly suspected^d^ | 259(4) | - | SMD 4.73 lower(6.86 lower to 2.61 lower) | ⨁◯◯◯Very low | IMPORTANT |
|  | Yes | serious^a^ | serious^b^ | not serious | not serious | publication bias strongly suspected^d^ | 672(9) | - | SMD 2.01 lower(2.68 lower to 1.33 lower) | ⨁◯◯◯Very low | IMPORTANT |
| FT3 | Yes | serious^a^ | serious^b^ | not serious | not serious | publication bias strongly suspected^d^ | 611(8) | - | SMD 0.83 SD higher(0.12 lower to 1.78 higher) | ⨁◯◯◯Very low | IMPORTANT |
| FT4 | Yes | serious^a^ | serious^b^ | not serious | not serious | publication bias strongly suspected^d^ | 611(8) | - | SMD 1.34 higher(0.59 higher to 2.08 higher) | ⨁◯◯◯Very low | IMPORTANT |
| TSH | Yes | serious^a^ | serious^b^ | not serious | not serious | publication bias strongly suspected^d^ | 672(9) | - | SMD 0.80 lower(1.71 lower to 0.11 higher) | ⨁◯◯◯Very low | IMPORTANT |
| TNF-α | Yes | serious^a^ | serious^b^ | not serious | not serious | publication bias strongly suspected^d^ | 230(3) | - | SMD 3.40 SD lower(5.66 lower to 1.14 lower) | ⨁◯◯◯Very low | NOT  IMPORTANT |
| IL-2 | Yes | serious^a^ | serious^b^ | not serious | serious^c^ | publication bias strongly suspected^d^ | 150(2) | - | SMD 2.31 lower(3.98 lower to 0.65 lower) | ⨁◯◯◯Very low | NOT IMPORTANT |
| IL-6 | Yes | serious^a^ | serious^b^ | not serious | serious^c^ | publication bias strongly suspected^d^ | 160(2) | - | MD 4.16 lower(6.17 lower to 2.15 lower) | ⨁◯◯◯Very low | NOT IMPORTANT |

a. The risk of bias is decreased by one level: There are some high risks and unclear risk bias, poor description of methodology including random sequence generation, allocation concealment, blinding and others.

b. The inconsistency is reduced by one level: I^2^≥ 50% for heterogeneity.

c. The inaccuracy is decreased by one level: Small sample sizes.

d. Too few studies.

# **Supplementary Fig. 1 Subgroup analysis of TPOAb (HT patients with normal thyroid function)**

(A) Intervention duration (T < 24week, T ≥ 24 weeks); (B) OS preparation (Bailing Capsule, Jinshuibao Capsule); (C) Different dose (dose ≤ 3 g/d, dose > 3 g/d).


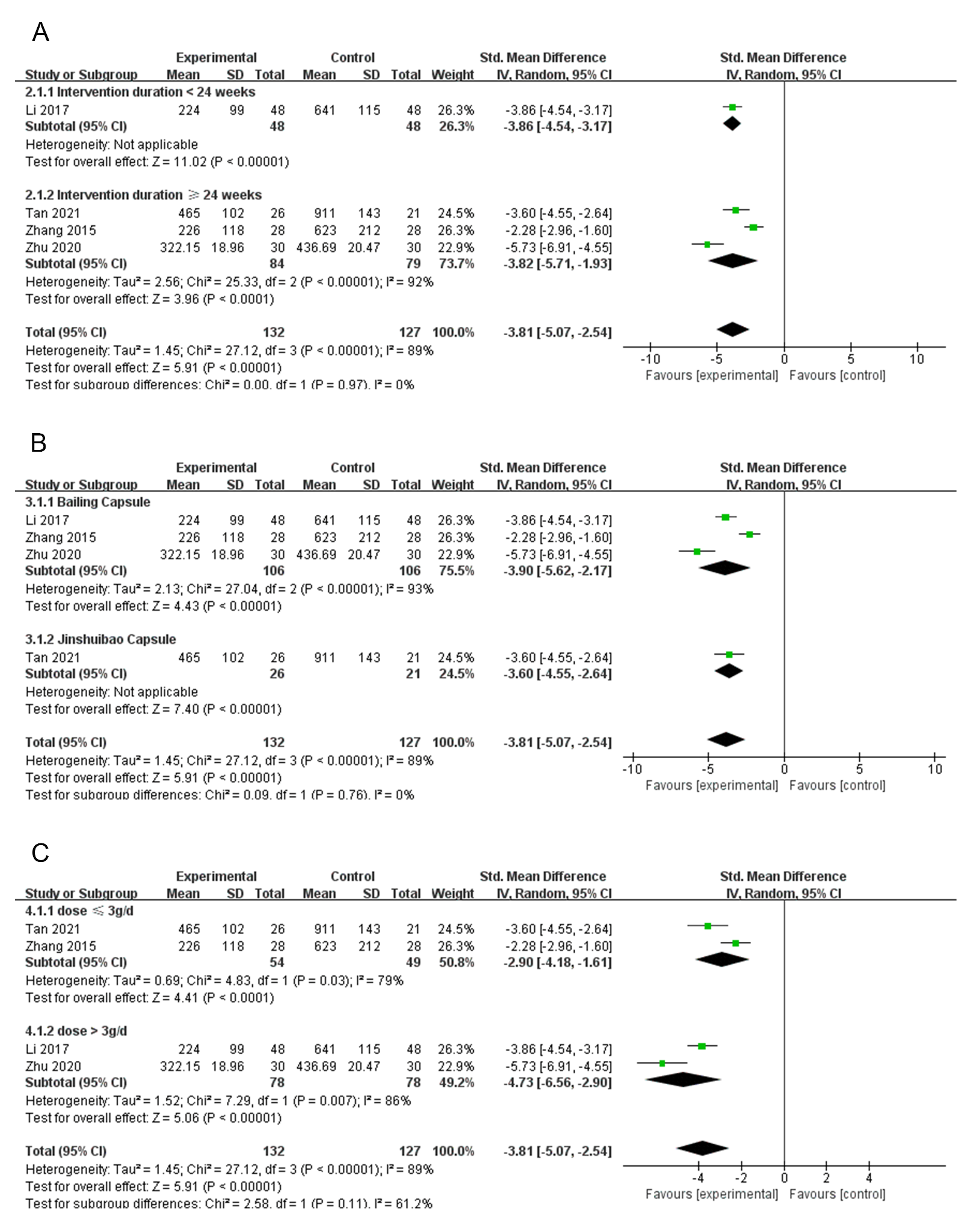


# **Supplementary Fig. 2 Subgroup analysis of TPOAb (HT patients with hypothyroidism)**

(A) Intervention duration (T < 24week, T ≥ 24 weeks); (B) OS preparation (Bailing Capsule, Jinshuibao Capsule); (C) Different dose (dose ≤ 3 g/d, dose > 3 g/d).


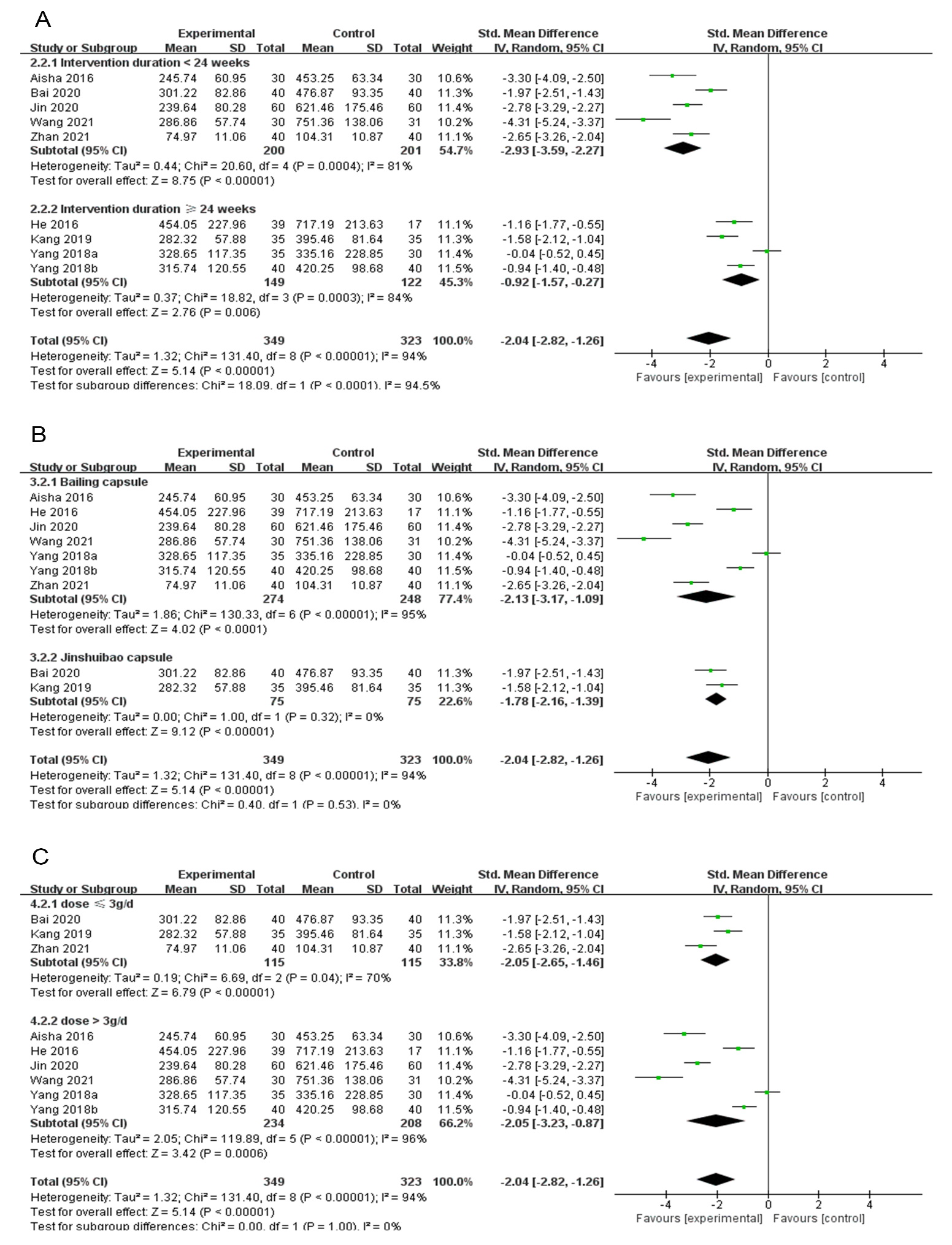


# **Supplementary Fig. 3 Subgroup analysis of TgAb (HT patients with normal thyroid function)**

(A) Intervention duration (T < 24week, T ≥ 24 weeks); (B) OS preparation (Bailing Capsule, Jinshuibao Capsule); (C) Different dose (dose ≤ 3 g/d, dose > 3 g/d).


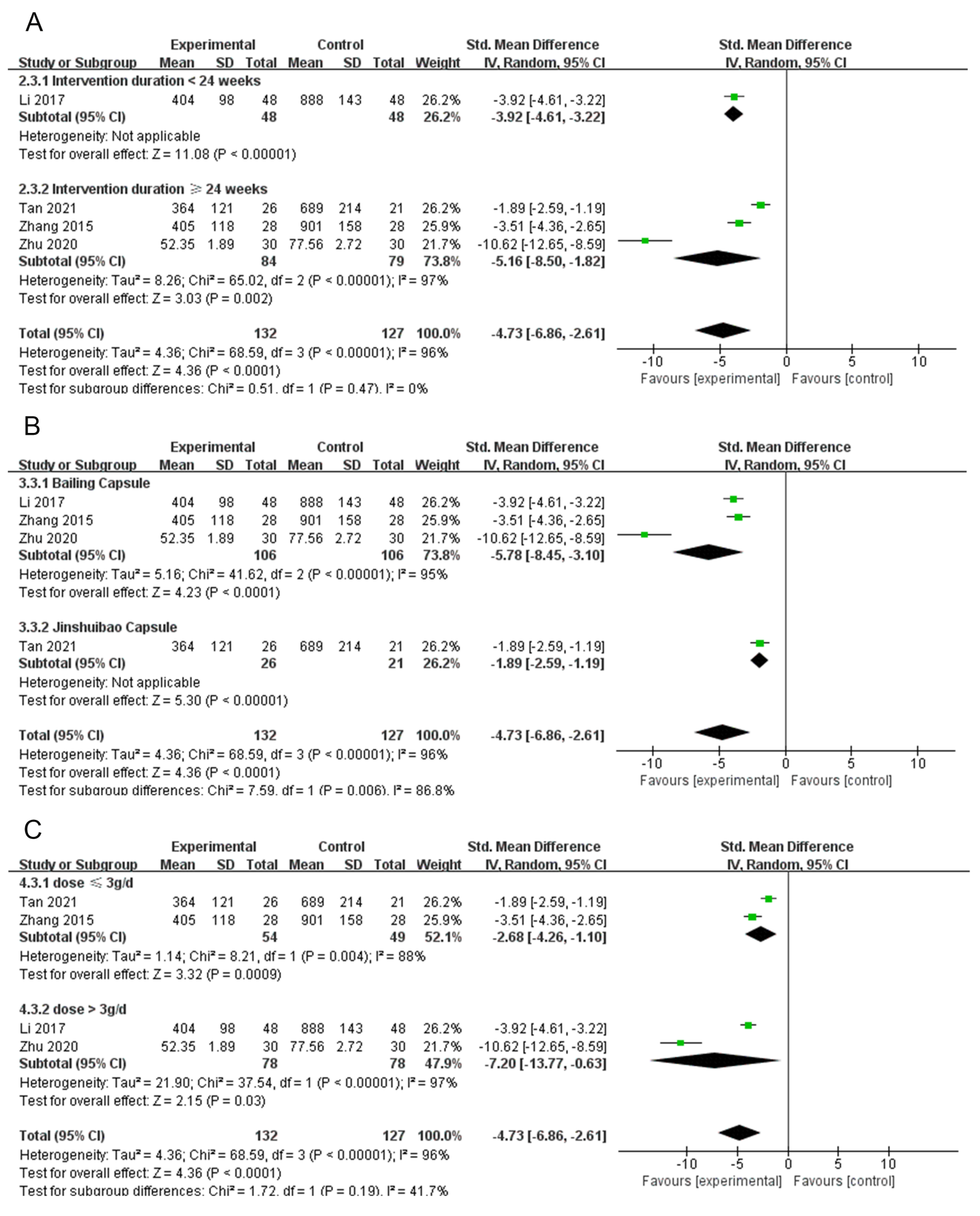


# **Supplementary Fig. 4 Subgroup analysis of TgAb (HT patients with hypothyroidism)**

(A) Intervention duration (T < 24week, T ≥ 24 weeks); (B) OS preparation (Bailing Capsule, Jinshuibao Capsule); (C) Different dose (dose ≤ 3 g/d, dose > 3 g/d).


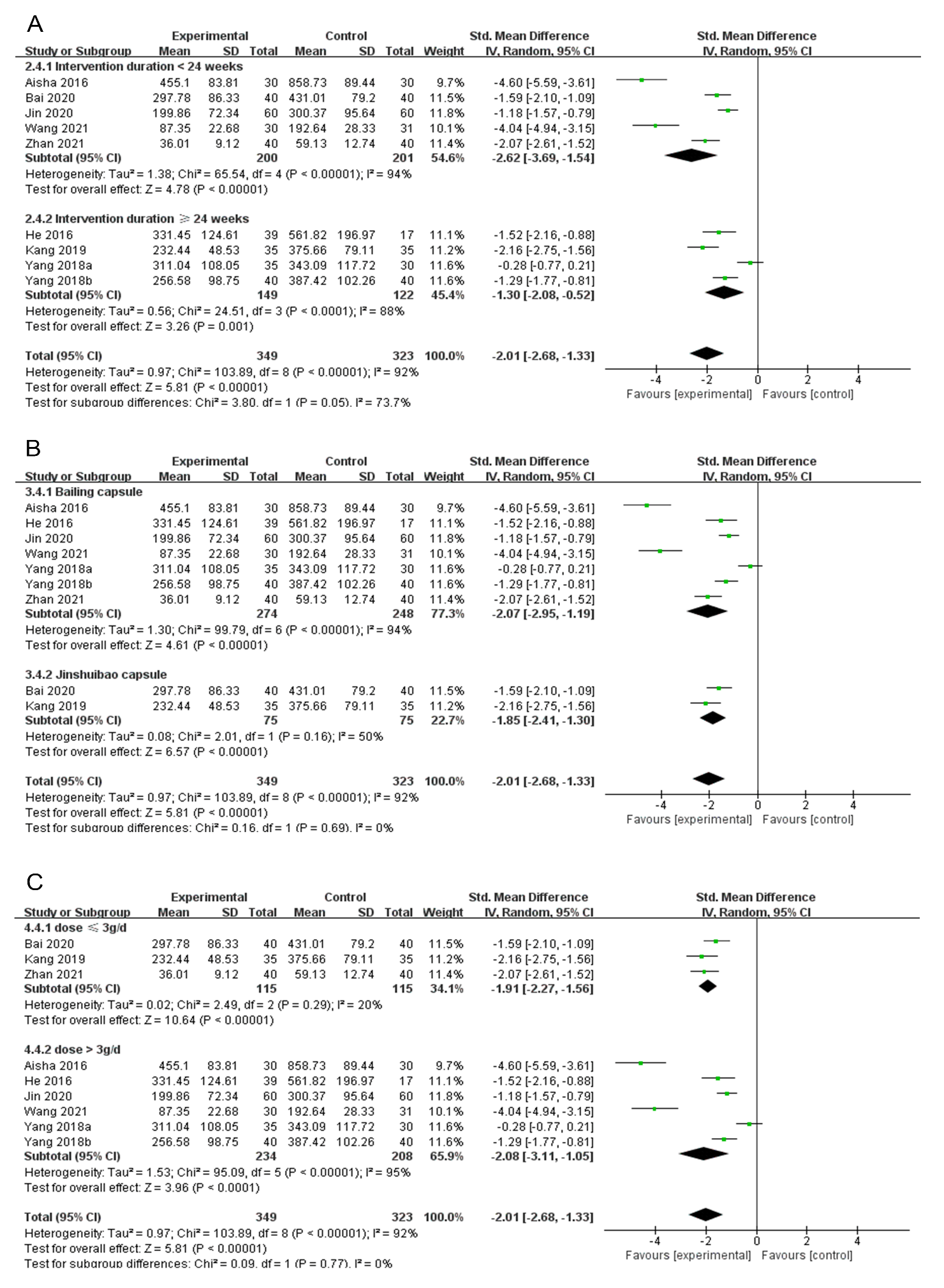


# Supplementary Fig. 5 **Subgroup analysis of FT3 (HT patients with hypothyroidism)**

(A) Intervention duration (T < 24week, T ≥ 24 weeks); (B) OS preparation (Bailing Capsule, Jinshuibao Capsule); (C) Different dose (dose ≤ 3 g/d, dose > 3 g/d).


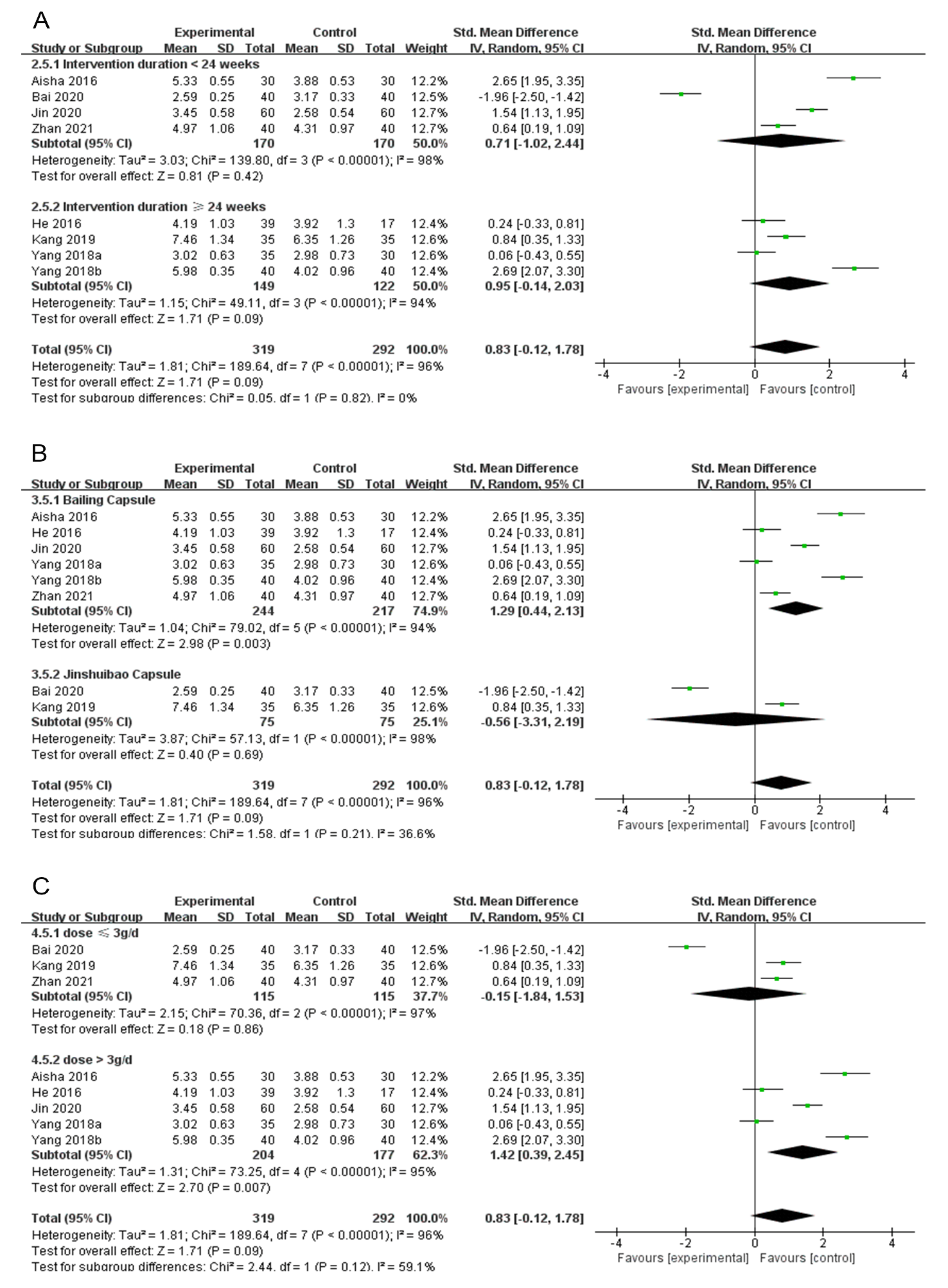


# Supplementary Fig. 6 **Subgroup analysis of FT4 (HT patients with hypothyroidism)**

(A) Intervention duration (T < 24week, T ≥ 24 weeks); (B) OS preparation (Bailing Capsule, Jinshuibao Capsule); (C) Different dose (dose ≤ 3 g/d, dose > 3 g/d).


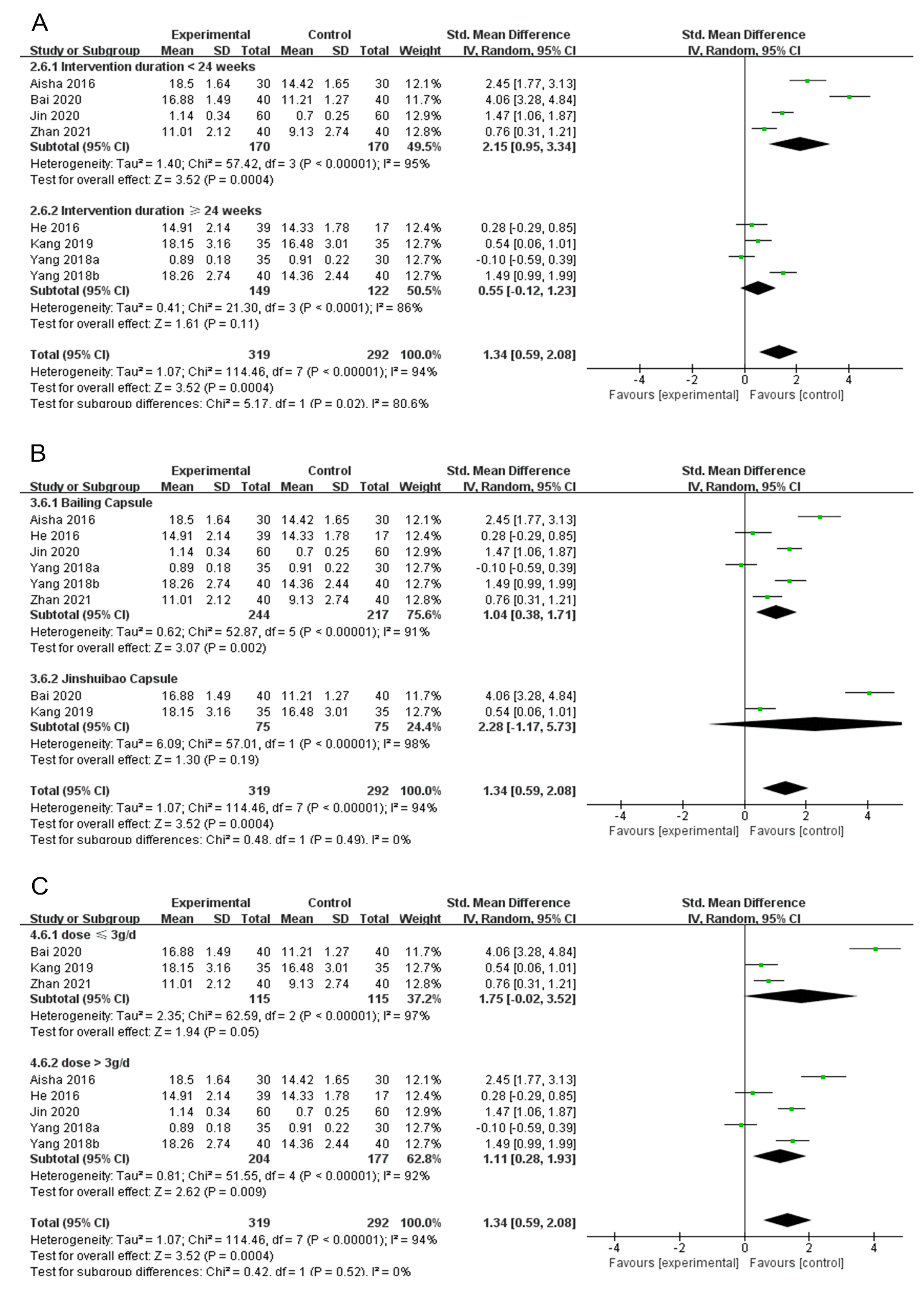


# Supplementary Fig. 7 **Subgroup analysis of TSH (HT patients with hypothyroidism)**

(A) Intervention duration (T < 24week, T ≥ 24 weeks); (B) OS preparation (Bailing Capsule, Jinshuibao Capsule); (C) Different dose (dose ≤ 3 g/d, dose > 3 g/d).


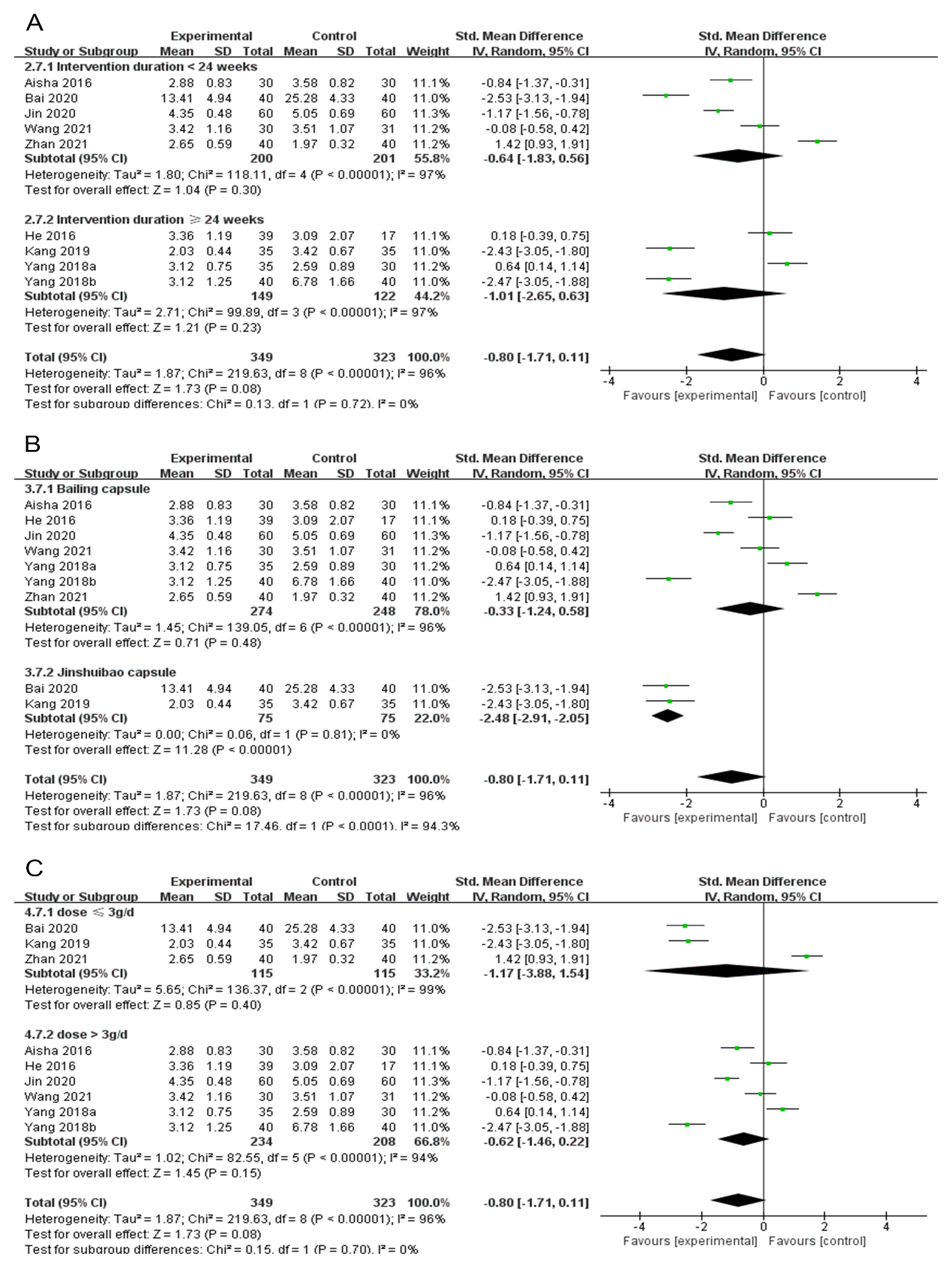


# Supplementary Fig. 8 **Subgroup analysis of** TNF-α

(A) Intervention duration (T < 24week, T ≥ 24 weeks); (B) OS preparation (Bailing Capsule, Jinshuibao Capsule).


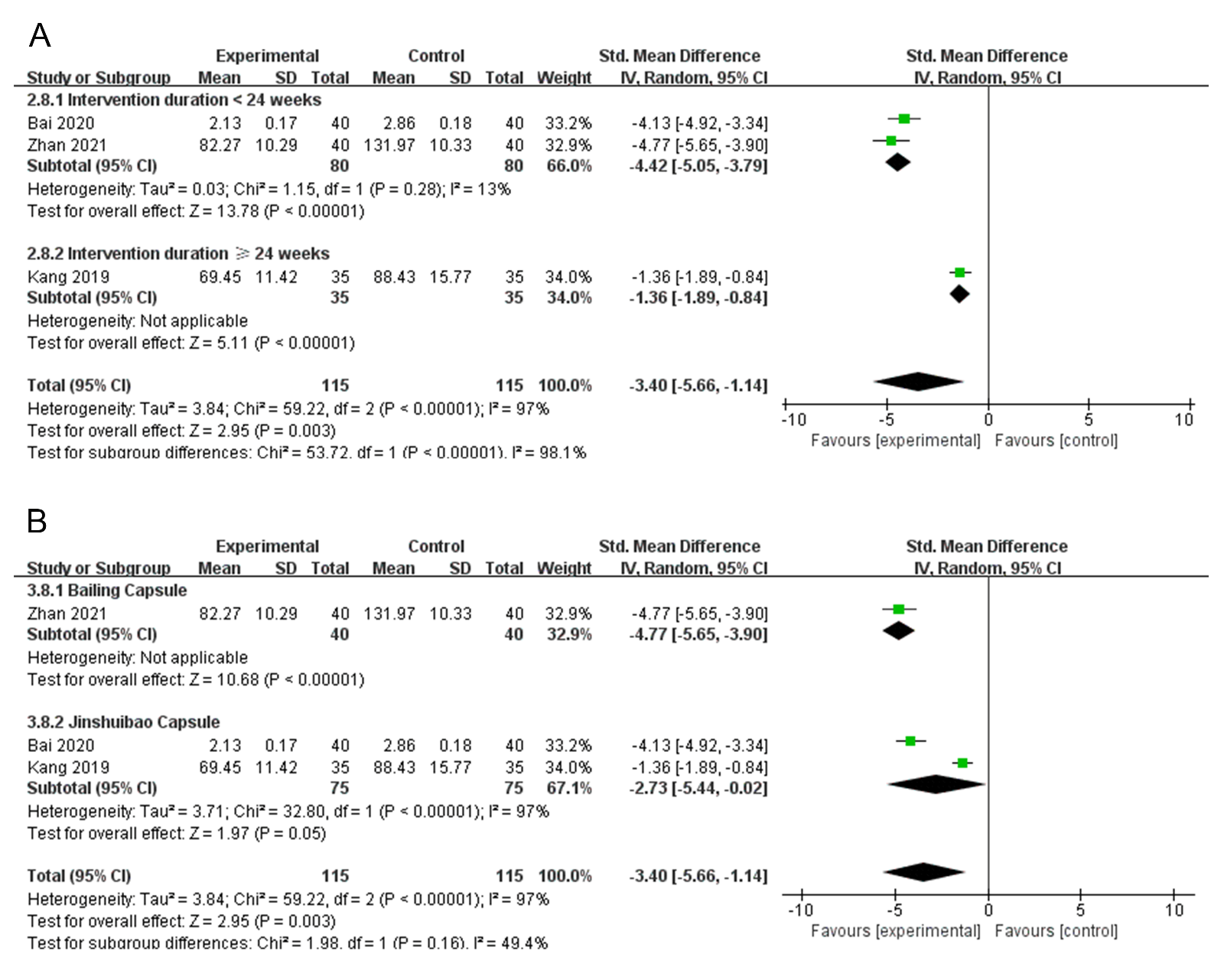

Supplement: Supplementary file 1 [file DataSheet1.zip › Additional files (revision-2)/Supplementary materials (revision-2).docx]
