# Supplementary material for: Efficacy and safety of Ophiocordyceps sinensis in the treatment of Hashimoto’s thyroiditis: a systematic review and meta-analysis
Source: Front Pharmacol. 2023 Oct 3;14:1272124. doi: 10.3389/fphar.2023.1272124 (PMC10579621; doi:10.3389/fphar.2023.1272124)
Supplement: Supplementary file 1 [file DataSheet1.zip › Additional files (revision-2)/Table1(revision).doc]

Table 1. Basic characteristics of included studies.

| Study ID | Sample size(E/C) | Age(year) | | Male/Female | | Intervention(s) | Comparators | Treatment duration(week) | With hypothyroidism | Outcomes |
| --- | --- | --- | --- | --- | --- | --- | --- | --- | --- | --- |
|  |  | E | C | E | C |  |  |  |  |  |
| Zhang, 2015 | 28/28 | 32.0±5.2 | | 3/53 | | BLC 0.4g tid + LID | LID | 24 | not | ①②③④⑤ |
| Li, 2017 | 48/48 | 48±5 | | 24/24 | 25/23 | BLC 2.5g tid + LID | LID | 12 | not | ①②③④⑤ |
| Zhu et al., 2020 | 30/30 | 35.07±1.50 | 34.73±1.45 | 0/30 | 0/30 | BLC 1.5g tid + LID | LID | 24 | not | ①②③④⑤ |
| Tan, 2021 | 26/21 | 28.0±5.7 | | 6/41 | | JSBC 1.5g tid + LID | LID | 28 | not | ①②③④⑤⑨ |
| Xue et al., 2021 | 40/40 | 41.47±4.15 | 41.25±4.13 | 20/20 | 18/22 | BLC 1.0g tid + LID | LID | 12 | not | ①③ |
| Aisha, 2016 | 30/30 | 40.26±8.17 | 40.71±8.35 | 1/29 | 1/29 | BLC 2.5g bid + LT4 | LT4 | 24 | yes | ①②③④⑤ |
| He et al., 2016 | 39/17 | 41.40±13.76 | 43.82±12.48 | 11/28 | 7/10 | BLC 2.0g tid + LT4 | LT4 | 24 | yes | ①②③④⑤ |
| Yang etal., 2018a | 35/30 | NA | | NA | NA | BLC 2.0g tid + LT4 | LT4 | 24 | yes | ①②③④⑤⑨ |
| Yang etal., 2018b | 40/40 | 35.5±9.8 | 35.9±11.2 | 8/32 | 7/33 | BLC 3.0g tid + LT4 | LT4 | 24 | yes | ①②③④⑤⑨ |
| Kang and Piao, 2019 | 35/35 | 47.14±8.83 | 48.32±9.12 | 3/32 | 5/30 | JSBC 0.99g tid + LT4 | LT4 | 24 | yes | ①②③④⑤⑥⑦⑨ |
| Bai etal., 2020 | 40/40 | 43.79±3.46 | 43.68±4.82 | 22/18 | 24/16 | JSBC 0.99g bid + LT4 | LT4 | 8 | yes | ①②③④⑤⑥⑧⑨ |
| Jin and Wang, 2020 | 60/60 | 43.28±6.87 | | 22/98 | | BLC 2.5g tid + LT4 | LT4 | 12 | yes | ①②③④⑤ |
| Wang et al., 2021 | 32/32 | 38.69±7.88 | 37.85±7.33 | 9/23 | 10/22 | BLC 2.5g tid + LT4 | LT4 | 12 | yes | ③④⑤ |
| Zhan and Chen, 2021 | 40/40 | 32.56±5.78 | 32.98±3.01 | 11/29 | 13/27 | BLC 1.0g tid + LT4 | LT4 | 8 | yes | ①②③④⑤⑥⑦⑧⑨ |

E: experimental group; C: control group; BLC: Bailing capsule; JSBC: Jinshuibao capsule; LID: low-iodine diet; LT4: levothyroxine; ①free triiodothyronine (FT3); ②free thyroxine (FT4); ③thyroid stimulating hormone (TSH); ④thyroid peroxidase antibody (TPOAb); ⑤thyroglobulin antibody (TgAb); ⑥tumor necrosis factor alpha (TNF-α); ⑦interleukin-2 (IL-2); ⑧interleukin-6 (IL-6); ⑨adverse events.
